# Sex hormones and the risk of myocardial infarction in women and men: a prospective cohort study in the UK Biobank

**DOI:** 10.1186/s13293-023-00546-3

**Published:** 2023-09-20

**Authors:** Katie Harris, Sanne A. E. Peters, Mark Woodward

**Affiliations:** 1 The George Institute for Global Health, University of New South Wales, Level 5, 1 King St, Newtown, Sydney, NSW 2042 Australia; 2 The George Institute for Global Health, Imperial College London, London, UK; 3 Julius Center for Health Sciences and Primary Care, University Medical Center Utrecht, Utrecht, The Netherlands

**Keywords:** Myocardial infarction, Sex hormones, UK Biobank, Women's health

## Abstract

**Objectives:**

There is conflicting evidence around the role of sex hormones with cardiovascular outcomes. The aim of this study was to examine the association of sex hormones with the risk of myocardial infarction (MI) in pre- and post-menopausal women, and men in the UK Biobank.

**Methods:**

The UK Biobank is a prospective population-based cohort study, that recruited over 500,000 (aged 40–69 years) women and men between 2006 and 2010.

Sex specific cox regression models, estimating hazard ratios (HRs) and women to men ratio of HRs (RHR) with respective 95% confidence intervals (CI), were used to model the association of sex hormones [oestrogen, testosterone, oestrogen: testosterone (O/T) ratio, sex hormone–binding globulin (SHBG) and the free androgen index (FAI)], measured at study baseline, with incident MI for women and men.

**Results:**

Data were from 479,797 participants [264,282 (55.1%) women] without a history of MI at study baseline. Over 12.5 years of follow-up, there were 4,908 MI events in women and 10,517 in men. Neither oestrogen nor testosterone were associated with MI in women and men after multiple adjustment. For men, but not women, a unit higher log-transformed O/T ratio was associated with a lower risk of MI 0.79 (0.65, 0.95) after adjustment for traditional CVD risk factors. The corresponding women to men RHR (95% CI) was 1.24 (0.99, 1.56). Higher SHBG (per unit) was also associated with a lower risk of MI in men 0.94 (0.89, 0.99), and not in women 1.02 (0.95, 1.09) after multiple adjustment, the corresponding women to men RHR (95% CI) was 1.09 (1.00, 1.18). Higher FAI was associated with a higher risk of MI in men 1.09 (1.02, 1.15), though not in women 0.97 (0.92, 1.02), the corresponding women to men RHR was 0.89 (0.82, 0.97). Finally, there were differential effects in the association of SHBG and FAI between pre- and post-menopausal women.

**Conclusions:**

A higher O/T ratio was associated with a lower risk of MI, and a higher FAI with a higher risk of MI after adjustment for CVD risk factors in men, but not in women. Thus, hormone ratios, rather than each alone, may play an important role in modulating the effect of MI.

**Supplementary Information:**

The online version contains supplementary material available at 10.1186/s13293-023-00546-3.

## Plain Language Summary

There are conflicting findings surrounding the association of sex hormones and myocardial infarction (MI) (heart disease). In particular, high oestradiol levels in women are often thought to be protective and explain why the rates of heart disease are lower in women than men. For men, those with low levels of testosterone are often thought to be more prone to develop heart disease in their lifetimes.

Our study presents a comprehensive analysis of the association of sex hormones (in isolation and also together via their ratios), in women and men using the large-scale UK Biobank.

We found that neither oestrogen nor testosterone alone were associated with heart disease in women and men after accounting for cardiovascular risk factors, but the ratio of testosterone and oestrogen was associated with a lower risk of heart disease in men, though not in women. We also saw the association of sex hormone–binding globulin (SHBG), and free androgen index (FAI) (calculated by the ratio of total testosterone level to SHBG) with heart disease was different between women and men, and between pre- and post-menopausal women.

This paper highlights the complex interplay between sex hormones with heart disease in the presence of age and cardiovascular risk factors. In particular the balance (ratio) of sex hormones maybe more important, rather than each in isolation, when exploring their association with heart disease.

**Supplementary Information:**

The online version contains supplementary material available at 10.1186/s13293-023-00546-3.

## Highlights

- Neither oestrogen nor testosterone were associated with MI in women and men after multiple adjustment.
- For men, but not women, higher oestrogen/testosterone ratio was associated with a lower risk of MI after adjustment for traditional CVD risk factors.
- Higher SHBG was associated with a lower risk of MI in men, but not women overall.
- Higher FAI was associated with a higher risk of MI in men but not women overall.
- There were differential effects in SHBG and FAI between pre- and post-menopausal women.

**Supplementary Information:**

The online version contains supplementary material available at 10.1186/s13293-023-00546-3.

## Introduction

Coronary heart disease (CHD), principally myocardial infarction (MI), is the leading cause of death for women and men globally [13]. There are, however, sex disparities, such that compared with age-matched men the incidence of CHD is lower and develops 7–10 years later in women [20]. Furthermore, mortality from CHD remains higher in men than women until old age [3].

There is inconsistency in the literature on the association of oestradiol and testosterone in women and men with CHD. One school of thought suggests higher levels of oestrogen might be associated with a lower risk of cardiovascular disease, such that oestrogen is thought to have a cardioprotective effect [14]. It is commonly assumed that high oestradiol levels in women explain, in part, why rates of CHD are lower in women than men. In particular, when there is a decline in oestrogen and a suggested increase in the risk of CHD this has been postulated to be as a result of the menopause transition [9]. However, more recently it has been shown that the association between oestrogen and MI is largely confounded by age, and further confounded by other cardiovascular risk factors [24], and menopause does not seem to be a causal factor for CHD risk [6]. While oestrogen levels are more commonly discussed in the context of women’s health, lower concentrations are found in men, and also play a critical role in men’s health, particularly in relation to the ratio of oestrogen with testosterone. Such that an imbalance in the sex hormones may be associated with an unfavourable cardiovascular disease risk profile [5].

There is a body of evidence that suggests that men with low levels of testosterone are more prone to develop coronary artery disease in their lifetimes [23]. However, studies have shown contrasting findings of no association and positive associations between testosterone levels and development of CHD [23]. Studies in women have also shown that lower concentrations of testosterone are associated with greater risk of CHD [28]; however, many studies are limited by sample size and only in postmenopausal women, excluding premenopausal women [7].

The association of sex hormone–binding globulin (SHBG), and free androgen index (FAI), calculated by the ratio of total testosterone level to SHBG, have also demonstrated conflicting findings in their associations with CHD in women and men [29, 32, 33, 33, 34, 34].

Further large-scale prospective epidemiological studies are needed to resolve the remaining gaps in our understanding of the cardiovascular effects of sex hormones. Therefore, this study seeks to scrutinise the association of sex hormones and their ratios [oestradiol, testosterone, oestradiol/testosterone (O/T) ratio, SHBG and FAI] with the risk of MI in pre- and post-menopausal women, and men in the UK Biobank.

## Methods

### Study population

The UK Biobank is a prospective population-based cohort study, that recruited over 500,000 women and men (aged 40–69 years) between 2006 and 2010. Individuals were invited to attend one of the 22 centres across the UK for baseline assessment, which included questionnaires soliciting information on lifestyle, medical history, and reproductive history. Physical measurements, and biochemical markers [urine, blood (packed red blood cells and serum)] in biological samples, were collected at baseline. Further details are provided in (https://biobank.ndph.ox.ac.uk/ukb/ukb/docs/biomarker_issues.pdf). Written informed consent was obtained for all UK Biobank participants electronically. UK Biobank has obtained Research Tissue Bank approval from its governing Research Ethics Committee, as recommended by the National Research Ethics Service. This research has been conducted using the UK Biobank Resource (Application No. 74018). Permission to use the UK Biobank Resource was approved by the access subcommittee of the UK Biobank Board.

### Outcome

The primary endpoint in this study was incident fatal or non-fatal MI, as defined by the UK Biobank Outcome Adjudication Group, using International Classification of Diseases (ICD)-10 codes: I21, I22, I23, I241, I252. Hospital inpatient data from England, Scotland, and Wales, and national death registers were used to identify the date of the first known MI after the date of baseline assessment. Follow-up for all participants started at the entry to the study up to 30th September 2021; or when fatal or non-fatal MI, or death was recorded. Participants with MI recorded before entry to the UK Biobank were excluded from these analyses (*n* = 12,042).

### Sex hormones

Oestradiol (pmol/L) was measured by two-step competitive analysis, testosterone (nmol/L) by one step competitive analysis, and SHBG (nmol/L) by two step sandwich immunoassay analysis on a Beckman Coulter Unicel Dxl 800, from serum samples collected at baseline.

Verification of assay and analyser performance was carried out using a rigorous protocol to assess the following parameters: precision, accuracy and bias, Linearity and Reportable range, including the limit of quantification, carryover, and multi-instrument comparison.

Where oestradiol and testosterone assay results were missing, the reason was collected by the UK Biobank (https://biobank.ndph.ox.ac.uk/ukb/field.cgi?id=30805 and https://biobank.ndph.ox.ac.uk/ukb/field.cgi?id=30855, Accessed 01/07/2022). If the readings were below the reportable limit, these low values were considered ‘naturally low’ rather than ‘missing’, to maximize the scientific utility of these data. UK Biobank conducted a series of robust and detailed biomarker assay and analyser quality procedures designed to minimize drift, bias and measurement uncertainty, and described in further detail elsewhere (https://biobank.ndph.ox.ac.uk/ukb/ukb/docs/serum_biochemistry.pdf). Thus, we were able to calculate whether oestradiol and testosterone levels were detectable, undetectable (original value below reportable limit) or missing (where a reason for missingness was recorded).

The O/T ratio was calculated by first converting oestradiol from pmol/L to nmol/L by dividing by 1000, then oestradiol (nmol/L) was divided by testosterone (nmol/L).

### Statistical analysis

Baseline data were summarised by sex, as median and interquartile interval (IQI) for continuous variables (due to the skewed nature of many of the sex hormones), and number and percentage for categorical variables.

Least squares means were used to model the sex-specific associations of MI by age category using binomial regression, to yield the average percentage of MI per age category. Sex specific associations of sex hormones by age category were obtained using least squares means with linear regression to yield average sex hormone level per age category. Age was categorised as < 45, 45–50, 50–55, 55–60, 60–65 and > 65 years. Age-adjusted least squares means, derived from linear regression for continuous variables and binomial regression for binary variables, were used to examine the sex specific association between CVD risk factors and O/T ratio levels.

Cox regression models, estimating hazard ratios (HRs) and 95% confidence intervals (CI), were used to model the association of sex hormones (oestradiol, testosterone, O/T ratio, SHBG and FAI) with MI for women and men. Sex-specific quarters for detectable levels of each hormone were calculated and modelled (including undetectable and missing) as the exposure of interest, where the lowest detectable quarter is considered the reference category. Separate analyses were conducted for models with for undetectable and missing levels, where detectable was the reference category. For comparisons involving more than two groups, CIs were estimated using floating absolute risks. Continuous associations were also explored for log transformed sex hormones, HR (95% CIs) were calculated per unit higher and per standard deviation higher (for comparability). Three sets of model adjustment were applied. First, crude (unadjusted) estimates were calculated; second, models were adjusted for age; and third, models were multiple adjusted for a prespecified set of covariates: age, smoking status, total cholesterol, BMI, systolic blood pressure, Townsend deprivation index (a measure of material deprivation), diabetes, any anti-hypertensive or lipid lowering medication. A further set of models were fit for oestradiol, testosterone and SBHG, adjusted for each other, using the three model adjustment strategies previously outlined.

Estimates for women and men compared using women to men ratio of hazard ratios (RHR) with 95% CI’s, calculated as the interaction term between each sex hormone measure and sex [31].

For women, baseline characteristics, analysis of rates of MI and levels of sex hormones by age group, and the association of sex hormones with MI were conducted by menopause status. A further subgroup analysis was conducted by menopause status and Hormone Replacement Therapy (HRT) use or not.

All analyses were performed using R Studio Version 4.2.0 (R Core Team, 2022).

## Results

There were 490,321 UK Biobank participants without a history of MI at study baseline. After further removal of 10,524 participants whose oestradiol and testosterone reason for missingness was not recorded 479,797 participants [264,282 (55.1%) women] were included in the present study. The median age at recruitment was 57 years for women and 58 for men (Table 1). Over 12.5 years of follow-up, there were 4908 MI events in women and 10,517 in men.

**Table 1:** Baseline characteristics and hormone levels by sex

Characteristics	Women	Men
*N* = 479,797	264,282	215,515
Oestradiol		
Detectable [*n* (%)]	57,904 (21.9)	17,669 (8.2)
Undetectable [*n* (%)]	177,202 (67.1)	174,347 (80.9)
Missing [*n* (%)]	29,176 (11.0)	23,499 (10.9)
Oestradiol [pmol/L]	396.7 (264.6, 634.4)	204.1 (188.8, 230.8)
Testosterone		
Detectable [*n* (%)]	210,584 (79.7)	203,986 (94.7)
Undetectable [*n* (%)]	39,692 (15.0)	218 (0.1)
Missing [*n* (%)]	14,006 (5.3)	11,311 (5.2)
Testosterone [nmol/L]	1.015 (0.723, 1.373)	11.648 (9.457, 14.164)
Oestradiol/testosterone (O/T ratio)		
Oestradiol and testosterone detectable [*n* (%)]	52,529 (19.9)	17,580 (8.2)
O/T ratio (nmol/L)	0.379 (0.237, 0.631)	0.016 (0.013, 0.021)
Sex hormone–binding globulin (SHBG)		
SHBG detectable [*n* (%)]	226,690 (85.8)	188,864 (87.6)
SHBG [nmol/L]	56.48 (40.05, 77.36)	36.93 (27.90, 48.18)
Free androgen index (FAI)		
FAI detectable [*n* (%)]	190,490 (72.1)	187,797 (87.1)
FAI nmol/L	1.80 (1.14, 2.85)	31.32 (25.21, 39.28)
Baseline characteristics and physical measures		
Age (years)	57.0 (50.0, 63.0)	58.0 (50.0, 63.0)
Systolic blood pressure (mmHg)	133.0 (121.0, 147.0)	139.5 (129.0, 151.5)
Current smoking [*n* (%)]	23,314 (8.8%)	26,578 (12.3%)
BMI kg/m^2^	26.09 (23.43, 29.68)	27.25 (24.94, 29.97)
Waist circumference (cm)	83.0 (75.0, 92.0)	96.0 (89.0, 103.0)
Waist to hip ratio %	81.25 (76.74, 86.32)	93.27 (89.22, 97.35)
Waist to height ratio %	51.10 (46.30, 56.89)	54.49 (50.76, 58.82)
Body fat %	36.7 (32.0, 41.3)	25.3 (21.5, 29.0)
Total cholesterol [mmol/L]	5.8 (5.1, 6.6)	5.5 (4.8, 6.2)
LDL cholesterol [mmol/L]	3.8 (3.0, 4.2)	3.5 (2.9, 4.1)
HDL cholesterol [mmol/L]	1.55 (1.32, 1.82)	1.24 (1.07, 1.46)
Diabetes, [*n* (%)]	9649 (3.7)	13,763 (6.4)
Townsend deprivation Index	− 2.16 (−3.65, 0.44)	−2.16 (−3.67, 0.57)

Numbers in table are median (interquartile interval)—unless otherwise indicated

*FAI* Free androgen index, *SHBG* sex hormone–binding globulin

**Fig. 1:**
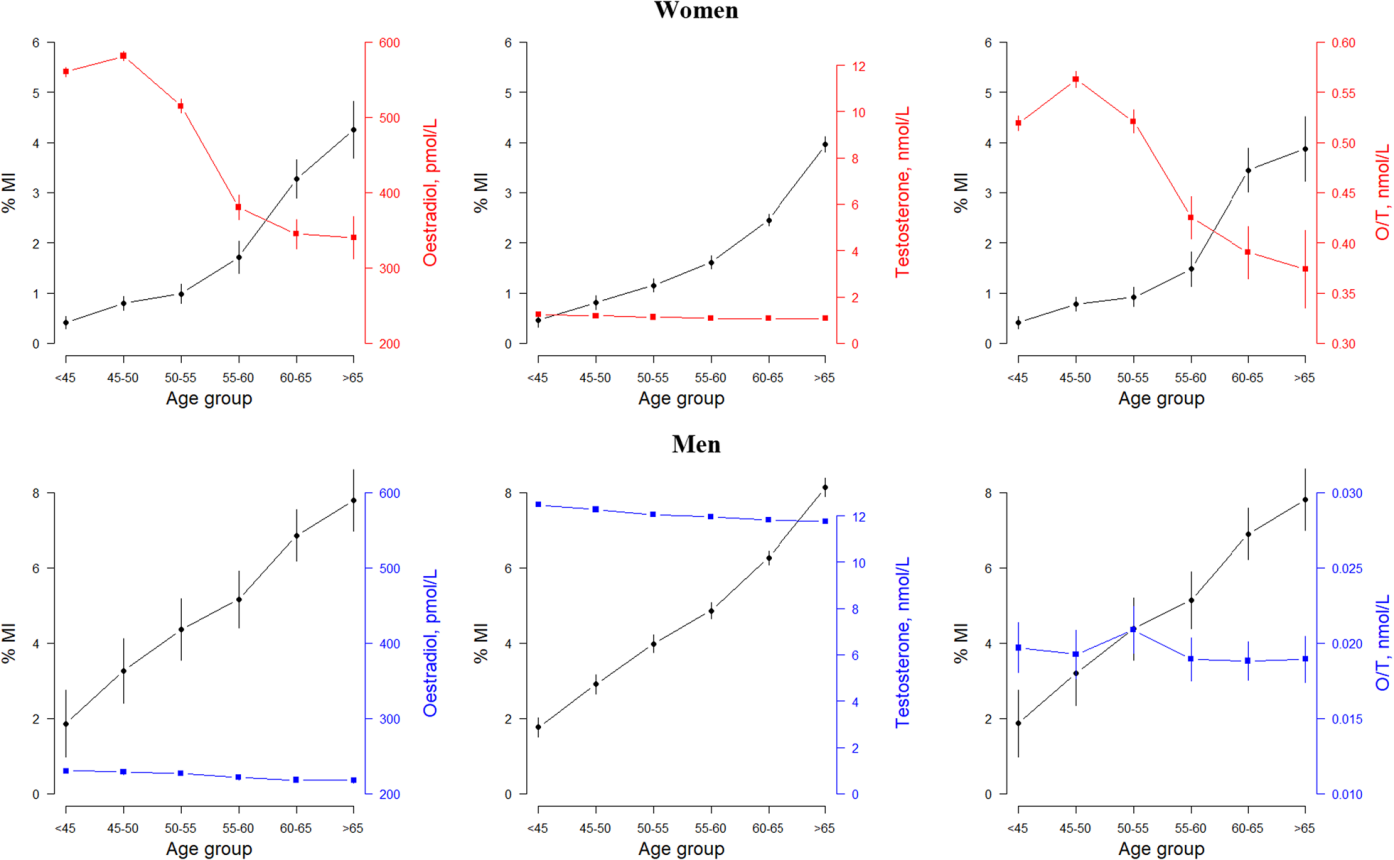
Rates of myocardial infarction and levels of sex hormones, with 95% confidence intervals, by sex and age group. *Blue* Men, *Pink/red* Women

Oestradiol was detectable in 21.9% of women (in 64.1% of premenopausal women and 5.3% of postmenopausal women) and in 8.2% of men (Additional file 1: Table S1). Testosterone was detectable in 79.7% of women (in 86.9% of premenopausal and 78.0% of postmenopausal women) and 94.7% of men. Women with detectable oestradiol levels were typically younger [Median (IQI)] 47 years (44, 51) than those with undetectable 60 years (55, 64) levels (Additional file 1: Table S2 and Fig. S1). Oestrogen levels for women with detectable levels were highest in the < 45, 45–50, 50–55 year age groups (560 pmol/L, 581 pmol/L, 515 pmol/L, respectively), and lower for those 55–60 years (381 pmol/L), and 60–65 years (345 pmol/L) (Fig. 1). Similar patterns were observed by menopause status (Additional file 1: Fig. S2 and S3).

Data for both oestradiol and testosterone, and thus the O/T were available in 19.9% of women and 8.2% of men. The median (IQI) O/T ratio was 0.379 (0.237, 0.631) nmol/L in women and 0.016 (0.013, 0.021) nmol/L in men (Additional file 1: Fig. S4). There were some variations in the age-adjusted mean risk factor levels according to quarters of O/T ratio (Additional file 1: Table S3).

SHBG was detectable in 85.8% of women and 87.6% of men. Median (IQI) SHBG levels were 56.5 (40.1, 77.4) nmol/L in women and 36.9 (27.9, 48.2) nmol/L in men. Corresponding levels were 62.4 (44.4, 84.8) nmol/L in premenopausal women and 54.9 (39.3, 74.5) nmol/L in postmenopausal women. FAI was detectable in 72.1% of women and 87.1% of men, median (IQI) levels were 1.80 (1.14, 2.85) nmol/L in women, and was similar by menopause status, and 31.32 (25.21, 39.28) nmol/L in men. SHBG was higher with higher age in men, compared with FAI that was lower with older age. Both SHBG and FAI tended to be lower with higher age in women (Additional file 1: Fig. S5).

### Associations of sex hormones with MI

#### Oestrogen

**Fig. 2:**
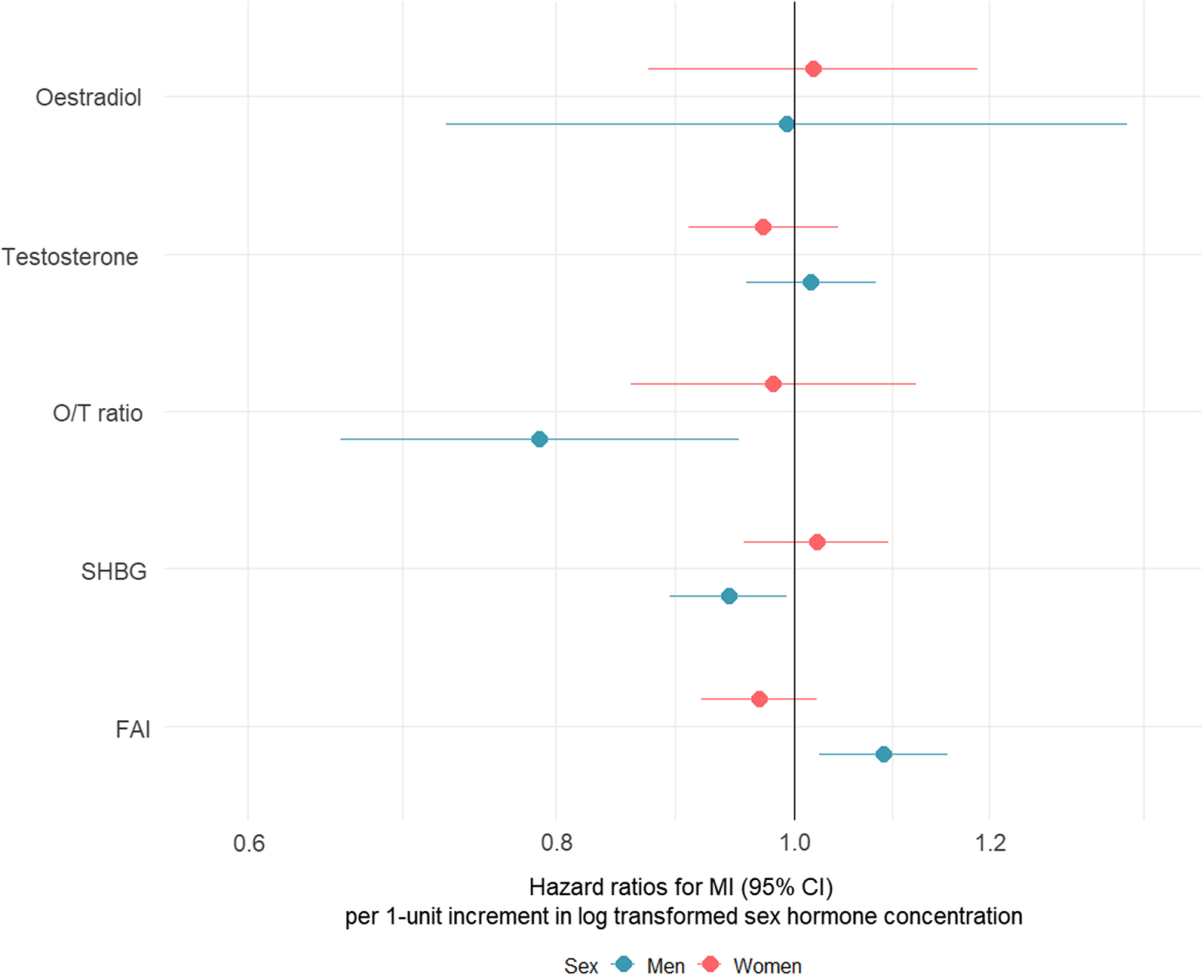
Association of per unit higher of log transformed sex hormones with MI by sex. Multiple adjusted hazard ratios (HR) with 95% confidence intervals: *Blue* Men, *Pink/red* Women. Multiple adjusted models adjusted for age, smoking status, total cholesterol, BMI, systolic blood pressure, Townsend deprivation score, diabetes, any anti-hypertensive or lipid lowering medication. O/T, oestradiol/testosterone; FAI, free androgen index, SHBG sex hormone–binding globulin

For women, in the unadjusted analyses, a unit higher in log-transformed oestradiol was associated with a lower risk MI: HR [95% confidence interval (CI)] 0.70 (0.60; 0.82) (Additional file 1: Table S4). After adjusting for age, the HR became 0.93 (0.79, 1.08), and after adjusting for traditional CVD risk factors, it was 1.02 (0.87, 1.19) (Fig. 2). Multivariable-adjusted HR (95% CI) for MI for undetectable levels of oestradiol compared with detectable levels were 0.94 (0.91, 0.98) for women and 0.96 (0.93, 0.98) for men.

#### Testosterone

For women, a unit higher in log-transformed testosterone was not associated with MI after multiple adjustment: HR (95% CI) 0.97 (0.91, 1.04). For men, in unadjusted and age adjusted analyses (per unit higher in log-transformed) testosterone was associated with a lower risk of MI: the corresponding HR’s (95% CI) were 0.72 (0.68, 0.76), 0.79 (0.75, 0.83), this no longer held after multiple adjustment 1.02 (0.946, 1.08).

#### O/T ratio

For men, (per unit higher in log-transformed) O/T ratio was associated with a lower risk of MI after adjusting for classical CVD risk factors HR (95% CI) 0.79 (0.65, 0.95), though not for women 0.98 (0.86, 1.12). The women to men RHR (95% CI) was [1.24 (0.99, 1.56)]. There was no significant linear trend in the HRs by quarters of O/T ratio for women *p* = 0.952, though there was for men (*p* = 0.050), The multiple adjusted HR (95% CI) for Q4 vs Q1 for women was 1.02 (0.83, 1.25), and for men 0.81 (0.70, 0.93) (Additional file 1: Fig. S6).

#### SHBG

For men, in the multivariable adjusted analyses, a unit higher in log-transformed SHBG was associated with a lower risk of MI HR (95% CI) 0.94 (0.89, 0.99), though not in women 1.02 (0.95; 1.09).

#### FAI

For men, in the multivariable adjusted analyses, a unit higher in log-transformed FAI was associated with a higher risk of MI: HR (95% CI) 1.09 (1.02, 1.15), though not in women 0.97 (0.92, 1.02), with a corresponding women to men RHR (95% CI) 0.89 (0.82, 0.97).

Analyses by quarters (Q4 vs Q1) of FAI yielded a HR (95% CI) of 1.13 (1.08, 1.19) for men and 0.92 (0.86, 0.99) for women, which yielded a women to men RHR (95% CI) 0.82 (0.75, 0.0.89).

#### Association of sex hormones after adjustment for each other

In models that adjusted for both sex hormones simultaneously, there remained no association between either oestrogen and testosterone with MI in women. In men, testosterone was associated with a higher risk of MI after adjustment for oestrogen and traditional CVD risk factors: HR (95% CI) 1.39 (1.12, 1.73) (Additional file 1: Table S5).

The association of SHBG with lower risk of MI in men remained after adjustment for testosterone 0.91 (0.86, 0.97), although not after adjustment for oestradiol 0.96 (0.80, 1.14), this, however, is likely as a consequence of the model being fitted using data of those with both detectable SHBG and oestradiol levels.

#### Associations of sex hormones with MI by menopause status

**Fig. 3:**
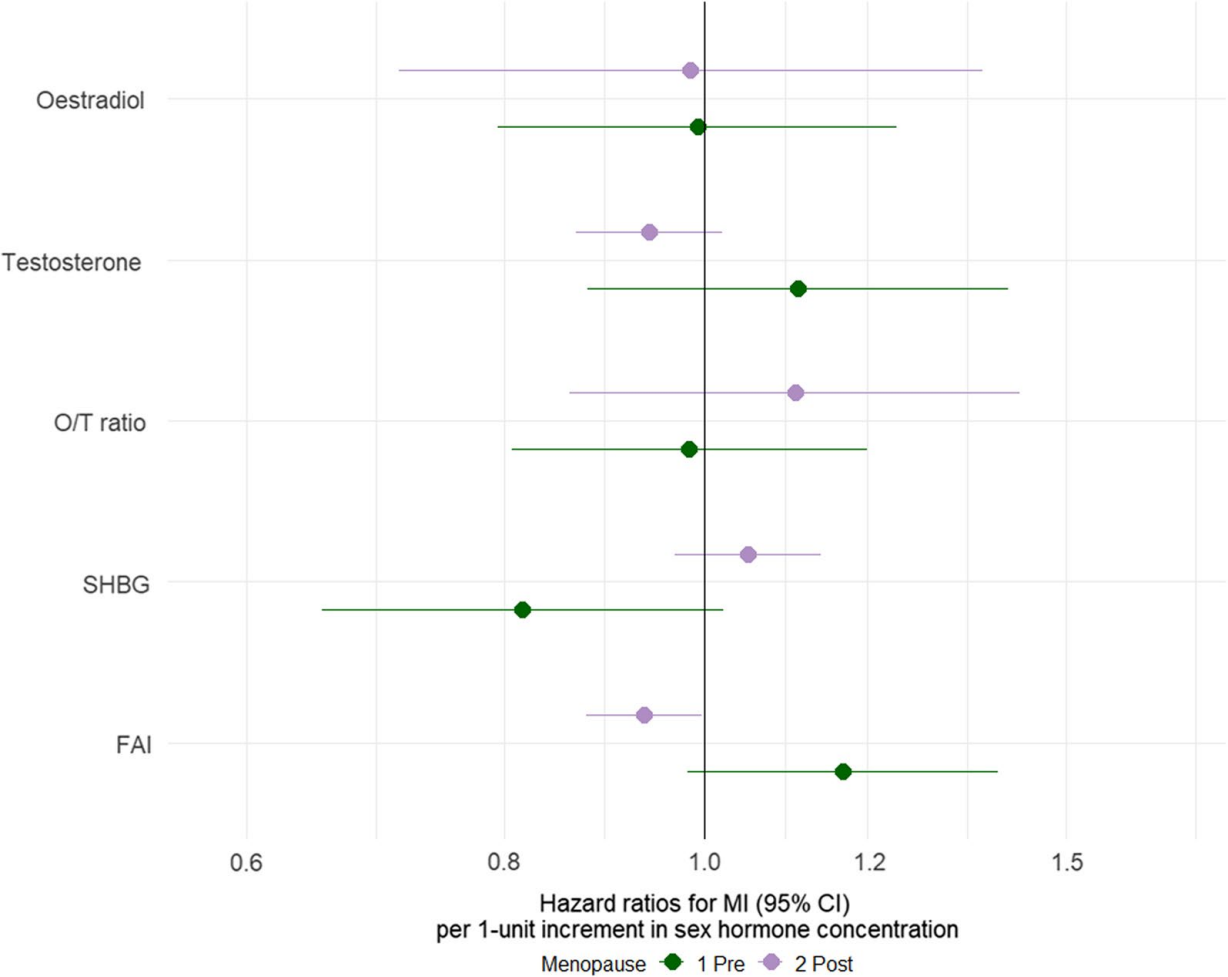
Association of per unit higher of log transformed sex hormones with MI by menopause status (for women). Multiple adjusted hazard ratios (HR) with 95% confidence intervals: *dark green* pre-menopause, *purple* post-menopause. Multiple adjusted hazard ratios (HR) with 95% confidence intervals: *dark green* pre-menopause, *purple* post-menopause. Multiple adjusted models adjusted for age, smoking status, total cholesterol, BMI, systolic blood pressure, Townsend deprivation score, diabetes, any anti-hypertensive or lipid lowering medication. O/T, oestradiol/testosterone; FAI, free androgen index; SHBG, sex hormone–binding globulin

There were no significant associations with MI per unit higher of log transformed oestradiol, testosterone and O/T ratio by menopause status, after adjusting for classical CVD risk factors. (Additional file 1: Table S6 and Fig. 3).

Higher FAI (Q4 vs Q1) was associated with a higher risk of MI in pre-menopausal women after multiple adjustment HR (95% CI) 1.37 (1.11, 1.68), but a lower risk in post-menopausal women 0.88 (0.81, 0.96), with a post- vs pre-menopausal women RHR (95% CI) of 0.64 (0.52, 0.80).

Higher SHBG (Q4 vs Q1) was associated with a lower risk of MI in pre-menopausal women 0.69 (0.53, 0.89), though not in post-menopausal women 0.99 (0.91, 1.08). This result yielded a post- vs pre-menopausal women RHR (95% CI) of 1.44 (1.10, 1.89) (Additional file 1: Fig. S7).

When conducted by HRT/not results were broadly similar to pre- and post-menopausal women overall (Additional file 1: Table S7). These results should be interpreted with caution due to the small number of events and detectable hormone levels among the groups.

## Discussion

This study assessed the association of sex hormones and their ratios with MI in women and men in the UK Biobank. Neither oestrogen nor testosterone alone were associated with MI in women and men after multiple adjustment. In men, but not women, higher O/T was associated with a lower risk of MI, after adjustment for traditional CVD risk factors. Higher FAI was associated with a higher risk of MI in men. There were differential effects by menopause status, such that being in the highest quarter of FAI vs the lowest quarter this conferred a higher risk of MI in premenopausal women, but a lower risk in post-menopausal women.. Higher SHBG was associated with a lower risk of MI in men with the effect being significantly lower in pre-menopausal women compared with post-menopausal women.

### Oestrogen

Oestrogen was not associated with MI in women and men after adjustment for CVD risk factors in this study. As shown in this study and previously reported, in unadjusted analyses a higher oestradiol was associated with a lower risk of MI in women, but this disappeared after age adjustment and further after multiple adjustment [24]. This finding suggests that the presumed cardioprotective effects of higher oestradiol are largely confounded by age and further by other CVD risk factors. Few studies have considered the role of oestradiol levels and the association of MI in men. Studies have shown that serum oestradiol level was significantly higher in the subjects with MI [16, 21, 25]. However, these studies did not consider the associations, and had limited and small sample sizes.

### Testosterone

In women, testosterone was not associated with MI in women after age and adjustment for multiple CVD risk factors. Total testosterone levels were associated with an increased risk of incident CVD and CHD in the study by [33, 34], though this study only considered post-menopausal women. In our analyses disaggregated by menopause status we saw no significant associations of testosterone with MI after multiple adjustment. Conflicting results from other studies in women that have shown that lower concentrations of testosterone were associated with greater risk of cardiovascular events [15, 28], but also that higher androgens in women influence abdominal adiposity and metabolic disorder, which in turn are risk factors for CVD [11].

Low testosterone has been shown to be associated with poorer health (diabetes, metabolic syndrome and obesity) in men [11, 17], which may explain why our association between testosterone and MI was explained after adjustment for traditional CVD risk factors in men. Our unadjusted and age adjusted results, in part, agree with other studies which have shown that men with low levels of testosterone are more prone to develop coronary artery disease in their lifetimes [23]. Our multiple adjusted results demonstrated no association between testosterone and MI, similar to another UK Biobank study [32]. Our present study builds upon this by including an extra 3 years of follow-up and including women, allowing for crucial comparisons between the sexes. Furthermore, corroborating our results is a study using Mendelian randomisation, which showed that serum testosterone predicted by nine genetic variants from the JMJD1C gene region in men from the UK Biobank was not associated with myocardial infarction (odds ratio: 1.17, 0.78–1.75) in men, nor women 0.91 (0.43–1.91) [19].

### O/T ratio

The MESA study demonstrated that higher testosterone/oestradiol ratio was associated with an increased incidence of CVD, and CHD events in post-menopausal women. [26, 33, 34]. We also saw corresponding findings that a higher O/T was associated with a lower risk of MI in women in unadjusted and age adjusted analyses. However, contrary to the results from MESA, our results after multiple adjustment, for all women and also when stratified by menopause status, the association did not remain.

In our study, a higher O/T was associated with a lower risk of MI after adjustment for traditional CVD risk factors in men. Thus, when testosterone is much larger and compared with oestrogen there is a higher risk of MI for men. We also saw that higher testosterone was associated with a higher risk of MI in men when including oestrogen in the model after multiple adjustment. This corresponds with Zheng et al. who state that it is the balance of testosterone and oestrogen, rather than the absolute levels in modulating the effect of androgens on CHD in males [5].

### SHBG

The highest levels of SHBG compared with the lowest levels conferred a lower risk of MI premenopausal in women in our study, but not postmenopausal women. The MESA study also showed that SHBG was not associated with CVD, CHD or Heart failure in post-menopausal women [33, 34]. Other studies have found no association between SHBG and incident CVD events in women. Data from ASPREE, saw no association between SHBG and cardiovascular events in fully adjusted models [15]. Data from the KORA-F4 cohort study [27], in peri and post-menopausal women and men, found that higher SHBG was associated with all-cause mortality events, but not specifically CVD mortality in both sexes.

Higher SHBG conferred a lower risk of MI in men in our study after adjustment for CVD risk factors. Inconsistent results have been reported previously for the association of SHBG and MI, though a growing body of evidence has also demonstrated the same directional relationship as we observe. A Mendellian randomisation study also showed genetically predicted higher SHBG was associated with lower risk of ischemic heart disease in men [35]. The previously mentioned study by Yeap and colleagues also showed men with lower SHBG concentrations have higher risk for MI in their analyses of UK Biobank data [32]. Participants with a larger number of cardiovascular risk factors had lower SHBG levels in the Rotterdam study [1] these authors also highlighted the importance of differences in the direction of the relationship after adjusting for age, which we also observed in our study.

### FAI

FAI is a measure of free testosterone level in the blood it is often used as baseline investigation for suspected hyperandrogenism in women, which may be as a result of polycystic ovary syndrome (PCOS). PCOS is a particular problem for women during their childbearing years and has been previously shown to contribute towards long term health problems, such as CVD [18]. This is pertinent, since we saw that FAI in the highest quarter compared with the lowest quarter was associated with a higher risk of MI in pre-menopausal women, and the converse in post-menopausal women.

The increased CVD events in women with PCOS have been attributed to increased adiposity and, possibly, an interaction with hyperandrogenism, although the mechanism has yet to be fully elucidated [11, 30].

Furthermore, a much smaller recent study in post-menopausal women did not see an association with FAI and MI [33, 34].

Levels of FAI were lower with higher age in men in this study. FAI (in the highest quarter compared with the lowest quarter) was also associated with a higher risk of MI in men after age adjustment and multiple adjustment. In unadjusted models the effect was the opposite. This highlights that such effects are strongly confounded by age and other CVD risk factors. Much smaller studies have previously shown that men with coronary artery disease had significantly lower levels of FAI [8, 10].

### Strengths and limitations

The strengths of this study include its prospective design with large sample size, including extensive blood biochemistry and biological samples for all participants. We investigated the association of several sex hormones and their ratios with MI in both women and men, where other studies have considered only women or men, thus not allowing for direct comparison. Our study also considers three adjustment strategies which enables scrutiny of the associations in unadjusted analysis, after age adjustment and after further adjustment for traditional CVD risk factors. There were, however, some limitations. Sex hormones in the UK Biobank were collected via immunoassays and thus maybe subject to inaccurate results [4]; however, the UK Biobank ensured all assays were conducted under systems designed for and consistent with the internationally recognised standard for testing and calibration laboratories. Second, oestradiol levels were detectable in 8.2% of men and 21.9% of women, which was skewed by menopause status (64.1% in premenopausal women and 5.5% in post-menopausal women); however, these are still considerably large sample sizes in comparison with other studies. Furthermore, the UK Biobank had a very low response rate to its baseline survey and participants are predominantly of white ethnicity and are relatively affluent individuals. However, with its large sample size and variety of exposures, risk factor associations in UK Biobank have been deemed to be widely generalisable [2, 12, 22]. Finally, the observational nature of this study does not allow for causal inferences.

### Perspectives and significance

This paper highlights the complex interplay between sex hormones with myocardial infarction particularly in the presence of age and cardiovascular risk factors. In particular the ratio of sex hormones that maybe more important, rather than each in isolation, when exploring their association with cardiovascular diseases.

### Supplementary Information

**Additional file 1: Table S1.** Baseline table by menopause status (women). **Table S2.** Age and number of incident MI events by sex specific quarters of sex hormone level. **Table S3.** Age-adjusted mean risk factor levels with 95% confidence interval according to O/T ratio and sex. **Table S4.** Association of sex hormones with MI by sex. **Table S5.** Association of combinations of sex hormones with MI by sex. **Table S6.** Association of sex hormones with MI by menopause status (for women). **Table S7.** Association of sex hormones with MI by menopause status and HRT use (for women). **Figure S1.** Percentage of Oestrogen and Testosterone detectable status by age group and sex. Pink bars represent women and blue represent men. **Figure S2.** Percentage of Oestrogen and Testosterone detectable status for women by age group and menopause status. Green bars represent premenopausal women and purple for post-menopausal women. **Figure S3.** Rates of myocardial infarction and levels of sex hormones, with 95% confidence intervals, by menopause status (for women) and age group. Footnote: Dark green lines represent sex hormone levels for pre-menopausal women and purple (dotted) for post-menopausal women. Black solid lines represent % MI by age group for pre-menopausal women and black dotted lines for post-menopausal women. Where lower confidence intervals were negative for the % MI these have not been plotted. O/T = Oestradiol/Testosterone, Free androgen index (FAI), sex hormone–binding globulin (SHBG). **Figure S4.** Plots of Oestradiol/Testosterone ratio (nmol/L) in women and men. Lower values of the O/T ratio were observed if oestrogen values were lower than testosterone values, and higher values if oestrogen was higher than testosterone. Of the 52,529 women with detectable levels of the O/T ratio, there were 5522 that had oestrogen concentration greater than testosterone concentration. In men the O/T ratio was generally low, since men tend to have a much higher testosterone level than oestrogen level. Of the 17,580 men with detectable levels of the O/T ratio, six had greater oestrogen concentration than their testosterone concentration. **Figure S5.** Rates of myocardial infarction and levels of SHBG, and free androgen index (FAI), with 95% confidence intervals, by sex and age group. Sex hormone–binding globulin (SHBG), Free androgen index (FAI) = Total Testosterone/SHBG x 100. **Figure S6.** Association of quarters of sex hormones with MI by sex. Multiple adjusted hazard ratios (HR) with 95% confidence intervals: *Blue* Men, *Pink/red* Women. Multiple adjusted models adjusted for age, smoking status, total cholesterol, BMI, Systolic blood pressure, Townsend deprivation score, diabetes, any anti-hypertensive or lipid lowering medication. O/T, Oestradiol/Testosterone; FAI, Free androgen index; SHBG, sex hormone–binding globulin. **Figure S7.** Association of quarters of sex hormones with MI by menopause status (for women). Multiple adjusted hazard ratios (HR) with 95% confidence intervals: *Dark green* pre menopause, *purple* post-menopause. Multiple adjusted models adjusted for age, smoking status, total cholesterol, BMI, Systolic blood pressure, Townsend deprivation score, diabetes, any anti-hypertensive or lipid lowering medication. O/T, Oestradiol/Testosterone; FAI, Free androgen index; SHBG, sex hormone–binding globulin.

## Data Availability

The data that support the findings of this study are available from the UK Biobank but restrictions apply to the availability of these data, which were used under license for the current study, and so are not publicly available. The UK Biobank resources are, however, available from the authors upon reasonable request and can be accessed through applications on their website (https://www.ukbiobank.ac.uk/).
