# Supplementary material for: Sex hormones and the risk of myocardial infarction in women and men: a prospective cohort study in the UK Biobank
Source: Biol Sex Differ. 2023 Sep 20;14:61. doi: 10.1186/s13293-023-00546-3 (PMC10510146; doi:10.1186/s13293-023-00546-3)
Supplement: Supplementary file 1 — Additional file 1: Table S1. Baseline table by menopause status (women). Table S2. Age and number of incident MI events by sex specific quarters of sex hormone level. Table S3. Age-adjusted mean risk factor levels with 95% confidence interval according to O/T ratio and sex. Table S4. Association of sex hormones with MI by sex. Table S5. Association of combinations of sex hormones with MI by sex. Table S6. Association of sex hormones with MI by menopause status (for women). Table S7. Association of sex hormones with MI by menopause status and HRT use (for women). Figure S1. Percentage of Oestrogen and Testosterone detectable status by age group and sex. Pink bars represent women and blue represent men. Figure S2. Percentage of Oestrogen and Testosterone detectable status for women by age group and menopause status. Green bars represent premenopausal women and purple for post-menopausal women. Figure S3. Rates of myocardial infarction and levels of sex hormones, with 95% confidence intervals, by menopause status (for women) and age group. Footnote: Dark green lines represent sex hormone levels for pre-menopausal women and purple (dotted) for post-menopausal women. Black solid lines represent % MI by age group for pre-menopausal women and black dotted lines for post-menopausal women. Where lower confidence intervals were negative for the % MI these have not been plotted. O/T = Oestradiol/Testosterone, Free androgen index (FAI), sex hormone–binding globulin (SHBG). Figure S4. Plots of Oestradiol/Testosterone ratio (nmol/L) in women and men. Lower values of the O/T ratio were observed if oestrogen values were lower than testosterone values, and higher values if oestrogen was higher than testosterone. Of the 52,529 women with detectable levels of the O/T ratio, there were 5522 that had oestrogen concentration greater than testosterone concentration. In men the O/T ratio was generally low, since men tend to have a much higher testosterone level than oestrogen level. Of the [file 13293_2023_546_MOESM1_ESM.pdf]

Additional File 1

Table S1: Baseline table by menopause status (women)

Hormones	No menopause (premenopausal)	Yes (postmenopausal)	Not sure (hysterectomy or other reason)
N (= 263,505) #	62,339	159,798	41,368
Age (years)	46.0 (43.0, 49.0)	61.0 (56.0, 64.0)	56.0 (51.0, 62.0)
Oestradiol			
Detectable (n (%))	39938 (64.1)	8503(5.3)	9234 (22.3)
Undetectable (n (%))	15500 (24.9)	133662 (83.6)	27574 (66.7
Missing (n (%))	6901(11.1)	17633 (11.0)	4560(11.0)
Oestradiol pmol/L	432.0 (290.3, 684.1)	281.8(213.1, 434.6)	369.0 (250.7, 603.8)
Testosterone			
Detectable (n (%))	54156 (86.9)	124650 (78.0)	31187 (75.4)
Undetectable (n (%))	4955 (7.9)	26613 (16.7)	7977 (19.3)
Missing (n (%))	3228 (5.2)	8535 (5.3)	2204 (5.3)
Testosterone nmol/L	1.13 (0.82, 1.49)	0.98 (0.70, 1.33)	0.97 (0.69, 1.32)
Oestradiol / Testosterone (O/T ratio) detectable	37470	7041	7820
O/T ratio (nmol/L)	0.40 (0.25, 0.65)	0.29 (0.18, 0.49)	0.38 (0.24, 0.64)
SHBG detectable	53100	137582	35336
SHBG nmol/L	62.4 (44.4, 84.8)	54.9 (39.3, 74.5)	54.6 (37.7, 77.2)
FAI detectable	48613	113238	28101
FAI nmol/L	1.81 (1.16, 2.86)	1.79 (1.14, 2.83)	1.77 (1.10, 2.90)
Ever taken hormone replacement therapy (n(%) yes)	2279 (3.7)	74751 (46.8)	22991 (55.6)

#Menopause status missing/not stated for 777 women

Table S2: Age and number of incident MI events by sex specific quarters of sex hormone level

Hormone	Q1	Q2	Q3	Q4	*Undetectable*	*Missing*	*Detectable*
**Women = 264,282**							
**Oestradiol (pmol/L)**	**175.0-264.6**	**264.7-396.7**	**396.8-634.4**	**634.5-14588.0**			
n	*14,483*	*14,472*	*14,473*	*14,476*	*177,202*	*29,176*	*57,904*
n (MI)	*186*	*117*	*99*	*114*	*3868*	*524*	*516*
Age(years)	*49 (44, 54)*	*47 (44, 51)*	*46 (43, 50)*	*47 (44, 50)*	*60 (55, 64)*	*57 (50, 63)*	*47 (44, 51)*
**Testosterone (nmol/L)**	**0.350-0.723**	**0.724-1.015**	**1.016-1.373**	**1.374-49.845**			
n	*52,747*	*52,704*	*52,496*	*52,637*	*39,692*	*14,006*	*210,584*
n (MI)	*1033*	*891*	*854*	*880*	*989*	*261*	*3658*
Age(years)	*59 (52, 63)*	*57 (50, 63)*	*56 (48, 62)*	*54 (47, 62)*	*60 (54, 64)*	*58 (50, 63)*	*57 (49, 62)*
**O/T (nmol/L)**	**0.00953 - 0.23699**	**0.23699 – 0.37886**	**0.37886– 0.63081**	**0.63083 – 38.78283**			
n	*13133*	*13132*	*13132*	*13132*			*52529*
n (MI)	*142*	*101*	*92*	*94*			*429*
Age(years)	*47 (43, 52)*	*47 (43, 51)*	*47 (43, 50)*	*47 (44, 50)*			*47 (44,51)*
**SHBG (nmol/L)**	**0.39-40.05**	**40.06-56.48**	**56.49-77.35**	**77.36-241.58**			
n	*56,689*	*56,682*	*56,646*	*56,673*	*721*	*36,871*	*226,690*
n (MI)	*1355*	*1082*	*981*	*788*	*10*	*692*	*4206*
Age(years)	*58 (51, 63)*	*58 (51, 63)*	*58 (50, 63)*	*56 (48, 62)*	*49 (43, 59)*	*57 (50, 63)*	*57 (50,63)*
**FAI (nmol/L)**	**0.16-1.14**	**1.14-1.80**	**1.80-2.86**	**2.86-146.33**			
n	*47,624*	*47,621*	*47,622*	*47,623*			*190,490*
n (MI)	*712*	*828*	*866*	*916*			*3322*
Age(years)	*57 (50, 63)*	*57 (49, 63)*	*57 (49, 63)*	*56 (49, 62)*			*57 (49, 63)*

Table S2 continued…..

Hormone	Q1	Q2	Q3	Q4	*Undetectable*	*Missing*	*Detectable*
**Men = 215,515**							
**Oestradiol (pmol/L)**	**175.0-188.8**	**188.9-204.1**	**204.2-230.9**	**230.0-4007.0**			
n	*4426*	*4429*	*4405*	*4409*	*174,347*	*23,499*	*17,669*
n (MI)	*224*	*236*	*239*	*208*	*8516*	*1094*	*907*
Age(years)	*58 (50, 64)*	*58 (50, 64)*	*58 (50, 63)*	*56 (49, 63)*	*58 (50, 63)*	*58 (50, 63)*	*58 (50, 63)*
**Testosterone (nmol/L)**	**0.354-9.457**	**9.458-11.648**	**11.649-14.164**	**14.165-54.342**			
n	*51,002*	*50,995*	*50,998*	*50,991*	*218*	*11,311*	*203,986*
n (MI)	*2876*	*2540*	*2351*	*2193*	*15*	*542*	*9960*
Age(years)	*59 (51, 64)*	*58 (50, 63)*	*58 (50, 63)*	*57 (49, 63)*	*65 (62, 67)*	*58 (50, 64)*	*58 (50, 63)*
**O/T (nmol/L)**	**0.00348 - 0.01320**	**0.01320 – 0.01635**	**0.01635 – 0.02078**	**0.02078 – 3.55752**			
n	*4395*	*4395*	*4395*	*4395*			*17580*
n (MI)	*221*	*215*	*234*	*232*			*902*
Age(years)	*57 (49, 64)*	*58 (50, 64)*	*58 (50, 64)*	*57 (49, 63)*			*58 (50, 63)*
**SHBG (nmol/L)**	**0.40-27.90**	**27.91-36.93**	**36.94-48.18**	**48.19-241.92**			
n	*47,236*	*47,216*	*47,219*	*47,193*	*11*	*26,640*	*188,864*
n (MI)	*2234*	*2296*	*2325*	*2358*	*0*	*1304*	*9213*
Age(years)	*53 (46, 60)*	*57 (49, 63)*	*59 (52, 64)*	*61 (55, 65)*	*64 (52, 67)*	*58 (50, 63)*	*58(50,63)*
**FAI (nmol/L)**	**0.40-25.12**	**25.12-31.20**	**31.20-39.13**	**39.13-1287.50**			
n	*46,950*	*46,949*	*46,949*	*46,949*			*187,797*
n (MI)	*2655*	*2416*	*2134*	*1945*			*9150*
Age(years)	*62 (57, 66)*	*60 (53, 64)*	*56 (49, 62)*	*51 (45, 58)*			*58 (50,63)*

O/T = Oestradiol / Testosterone, Free androgen index (FAI), sex hormone–binding globulin (SHBG), Age(years) presented as median (IQI)

Table S3: Age-adjusted mean risk factor levels with 95% confidence interval according to O/T ratio and sex

O/T ratio quarters	Women Q1	Women Q2	Women Q3	Women Q4	Men Q1	Men Q2	Men Q3	Men Q4
Systolic Blood Pressure (mmHg)	129.13 (128.86, 129.41)	127.77 (127.50, 128.05)	127.02 (126.75, 127.29)	126.19 (125.91, 126.46)	139.27 (138.76, 139.78)	140.58 (140.07, 141.09)	142.07 (141.56, 142.58)	143.22 142.71, 143.73
Current smoking %	12.23 (11.71, 12.76)	10.70 (10.18, 11.22)	9.63 (9.11, 10.15)	8.59 (8.07, 9.11)	16.31 (15.33, 17.28)	11.97 (11.00, 12.95)	10.85 (9.87, 11.82)	11.35 (10.37, 12.33)
BMI kg/m^2	27.80 (27.71, 27.89)	26.85 (26.76, 26.94)	26.45 (26.36, 26.54)	25.84 (25.74, 25.93)	26.66 (26.52, 26.79)	27.66 (27.52, 27.79)	28.67 (28.54, 28.81)	30.41 (30.27, 30.55)
Waist circumference (cm)	85.32 (85.11, 85.53)	83.22 (83.01, 83.43)	82.23 (82.01, 82.44)	80.81 (80.60, 81.03)	93.56 (93.21, 93.91)	96.21 (95.85, 96.56)	99.00 (98.65, 99.35)	103.50 (103.15, 103.85)
Waist to hip ratio %	81.06 (80.94, 81.17)	80.45 (80.33, 80.57)	80.11 (79.99, 80.23)	79.50 (79.38, 79.62)	91.81 (91.62, 92.00)	93.07 (92.88, 93.26)	94.18 (93.99, 94.37)	95.97 (95.78, 96.16)
Waist to height ratio %	52.16 (52.03, 52.30)	50.86 (50.73, 50.99)	50.29 (50.16, 50.43)	49.39 (49.26, 49.52)	53.31 (53.11, 53.51)	54.76 (54.56, 54.96)	56.28 (56.08, 56.48)	58.90 (58.70, 59.10)
Body fat %	36.53 (36.41, 36.65)	35.37 (35.25, 35.49)	34.80 (34.68, 34.92)	33.86 (33.73, 33.98)	23.56 (23.38, 23.73)	24.85 (24.67, 25.02)	26.19 (26.02, 26.37)	28.01 (27.84, 28.19)
Total Cholesterol mmol/L	5.51 (5.49, 5.53)	5.48 (5.47, 5.50)	5.44 (5.42, 5.45)	5.37 (5.36, 5.39)	5.44 (5.41, 5.47)	5.43 (5.40, 5.47)	5.40 (5.37, 5.43)	5.31 (5.28, 5.35)
LDL Cholesterol mmol/L	3.40 (3.39, 3.41)	3.36 (3.35, 3.37)	3.31 (3.30, 3.32)	3.24 (3.23, 3.25)	3.45 (3.42, 3.47)	3.46 (3.44, 3.49)	3.44 (3.41, 3.46)	3.36 (3.34, 3.39)
HDL Cholesterol mmol/L	1.53 (1.52, 1.53)	1.55 (1.55, 1.56)	1.57 (1.56, 1.58)	1.59 (1.58, 1.60)	1.36 (1.36, 1.37)	1.30 (1.29, 1.31)	1.26 (1.25, 1.27)	1.21 (1.21, 1.22)
Diabetes %	2.77 (2.52, 3.02)	2.25 (2.00, 2.50)	2.08 (1.83, 2.33)	1.70 (1.45, 1.95)	4.55 (3.80, 5.30)	5.61 (4.86, 6.36)	7.06 (6.31, 7.81)	10.66 (9.91, 11.41)
Townsend Deprivation Index	-1.01 (-1.07, -0.96)	-1.12 (-1.17, 1.07)	-1.15 (-1.20, -1.10)	-1.26 (-1.31, -1.21)	-0.84 (-0.94, -0.75)	-1.17 (-1.27, -1.08)	-1.22 (-1.32, -1.13)	-1.11 (-1.20, -1.01)

O/T = Oestradiol / Testosterone

Table S4: Association of sex hormones with MI by sex

	Women	Women	Women	Men	Men	Men	Women to Men RHR	Women to Men RHR	Women to Men RHR
	**Unadjusted**	**Age-adjusted**	**Multiple adjusted**	**Unadjusted**	**Age-adjusted**	**Multiple adjusted**	**Unadjusted**	**Age-adjusted**	**Multiple adjusted**
**Oestradiol pmol/L**									
Q1	1.00 (0.87, 1.15)	1.00 (0.87, 1.16)	1.00 (0.86, 1.16)	1.00 (0.88, 1.14)	1.00 (0.88, 1.14)	1.00 (0.88, 1.14)	1.00 (0.82, 1.21)	1.00 (0.82, 1.22)	1.00 (0.82, 1.22)
Q2	0.63 (0.52, 0.75)	0.78 (0.65, 0.94)	0.86 (0.71, 1.03)	1.06 (0.93, 1.20)	1.05 (0.92, 1.19)	1.06 (0.93, 1.21)	0.59 (0.48, 0.74)	0.74 (0.60, 0.93)	0.81 (0.65, 1.01)
Q3	0.53 (0.44, 0.65)	0.73 (0.60, 0.89)	0.82 (0.67, 1.01)	1.08 (0.95, 1.23)	1.08 (0.95, 1.22)	1.05 (0.92, 1.19)	0.49 (0.39, 0.62)	0.67 (0.53, 0.85)	0.78 (0.62, 0.99)
Q4	0.61 (0.51, 0.74)	0.89 (0.74, 1.08)	1.05 (0.87, 1.27)	0.93 (0.81, 1.07)	0.98 (0.85, 1.12)	0.93 (0.81, 1.07)	0.66 (0.52, 0.83)	0.92 (0.73, 1.15)	1.13 (0.90, 1.43)
Log transformed (Per unit higher)	0.70 (0.60, 0.82)	0.93 (0.79, 1.08)	1.02 (0.87, 1.19)	0.99 (0.74, 1.34)	1.14 (0.85, 1.55)	0.99 (0.72, 1.36)	0.71 (0.50, 0.99)	0.81 (0.58, 1.13)	1.02 (0.72, 1.46)
Log transformed (Per SD (0.62) higher)	0.80 (0.73, 0.88)	0.95 (0.87, 1.05)	1.01 (0.92, 1.11)	1.00 (0.83, 1.20)	1.09 (0.90, 1.31)	1.00 (0.82, 1.21)	0.81 (0.65, 0.99)	0.88 (0.71, 1.08)	1.01 (0.81, 1.26)
Detectable	1.00 (0.92, 1.09)	1.00 (0.91, 1.10)	1.00 (0.90, 1.10)	1.00 (0.94, 1.07)	1.00 (0.94, 1.07)	1.00 (0.94, 1.07)	1.00 (0.90, 1.11)	1.00 (0.89, 1.12)	1.00 (0.89, 1.13)
Undetectable	2.47 (2.39, 2.55)	1.00 (0.97, 1.04)	0.94 (0.91, 0.98)	0.95 (0.93, 0.97)	0.95 (0.93, 0.97)	0.96 (0.93, 0.98)	2.61 (2.51, 2.71)	1.06 (1.02, 1.10)	0.99 (0.95, 1.03)
Missing	2.05 (1.88, 2.23)	0.97 (0.89, 1.06)	0.90 (0.80, 1.00)	0.91 (0.86, 0.97)	0.91 (0.86, 0.97)	0.92 (0.85, 0.99)	2.24 (2.02, 2.49)	1.07 (0.96, 1.18)	0.98 (0.85, 1.12)
**Testosterone nmol/L**									
Q1	1.00 (0.94, 1.06)	1.00 (0.94, 1.06)	1.00 (0.94, 1.06)	1.00 (0.96, 1.04)	1.00 (0.96, 1.04)	1.00 (0.96, 1.04)	1.00 (0.93, 1.07)	1.00 (0.93, 1.07)	1.00 (0.93, 1.08)
Q2	0.86 (0.81, 0.92)	0.92 (0.87, 0.99)	0.91 (0.85, 0.97)	0.88 (0.85, 0.92)	0.91 (0.87, 0.94)	1.03 (0.99, 1.07)	0.98 (0.91, 1.06)	1.02 (0.94, 1.10)	0.89 (0.82, 0.96)
Q3	0.83 (0.78, 0.89)	0.95 (0.89, 1.02)	0.90 (0.84, 0.97)	0.81 (0.78, 0.85)	0.85 (0.81, 0.88)	1.02 (0.98, 1.06)	1.02 (0.94, 1.10)	1.13 (1.04, 1.22)	0.89 (0.82, 0.96)
Q4	0.85 (0.80, 0.91)	1.04 (0.97, 1.11)	0.93 (0.87, 1.00)	0.76 (0.73, 0.79)	0.81 (0.78, 0.85)	1.03 (0.99, 1.08)	1.12 (1.04, 1.21)	1.28 (1.18, 1.38)	0.90 (0.83, 0.98)
Log transformed (Per unit higher)	0.91 (0.85, 0.97)	1.06 (0.99, 1.13)	0.97 (0.91, 1.04)	0.72 (0.68, 0.76)	0.79 (0.75, 0.83)	1.02 (0.96, 1.08)	1.26 (1.15, 1.37)	1.34 (1.23, 1.46)	0.96 (0.87, 1.05)
Log transformed (Per SD (1.28) higher)	0.88 (0.81, 0.96)	1.07 (0.98, 1.17)	0.96 (0.88, 1.05)	0.66 (0.61, 0.71)	0.74 (0.69, 0.79)	1.02 (0.94, 1.10)	1.34 (1.20, 1.50)	1.45 (1.30, 1.63)	0.94 (0.84, 1.06)
Detectable	1.00 (0.97, 1.03)	1.00 (0.97, 1.03)	1.00 (0.97, 1.03)	1.00 (0.98, 1.02)	1.00 (0.98, 1.02)	1.00 (0.98, 1.02)	1.00 (0.96, 1.04)	1.00 (0.96, 1.04)	1.00 (0.96, 1.04)
Undetectable	1.44 (1.36, 1.54)	1.20 (1.13, 1.28)	1.25 (1.17, 1.33)	1.45 (0.87, 2.40)	1.02 (0.61, 1.68)	0.90 (0.54, 1.49)	1.00 (0.60, 1.66)	1.18 (0.71, 1.97)	1.39 (0.84, 2.32)
Missing	1.10 (0.97, 1.24)	1.06 (0.94, 1.19)	0.91 (0.67, 1.26)	1.00 (0.92, 1.09)	1.00 (0.92, 1.08)	1.11 (0.91, 1.34)	1.09 (0.94, 1.26)	1.06 (0.91, 1.23)	0.83 (0.57, 1.20)
**O/T ratio nmol/L**									
Q1	1.00 (0.85, 1.18)	1.00 (0.85, 1.18)	1.00 (0.84, 1.19)	1.00 (0.88, 1.14)	1.00 (0.88, 1.14)	1.00 (0.87, 1.15)	1.00 (0.81, 1.23)	1.00 (0.81, 1.24)	1.00 (0.80, 1.25)
Q2	0.71 (0.58, 0.86)	0.81 (0.67, 0.98)	0.92 (0.76, 1.12)	0.97 (0.85, 1.11)	0.94 (0.82, 1.07)	0.91 (0.79, 1.04)	0.73 (0.58, 0.93)	0.86 (0.68, 1.09)	1.01 (0.80, 1.28)
Q3	0.65 (0.53, 0.79)	0.76 (0.62, 0.94)	0.89 (0.73, 1.10)	1.06 (0.93, 1.20)	1.03 (0.91, 1.17)	0.91 (0.80, 1.04)	0.61 (0.48, 0.78)	0.74 (0.58, 0.94)	0.98 (0.77, 1.25)
Q4	0.66 (0.54, 0.81)	0.80 (0.66, 0.98)	1.02 (0.83, 1.25)	1.05 (0.92, 1.19)	1.05 (0.92, 1.19)	0.81 (0.70, 0.93)	0.63 (0.50, 0.81)	0.77 (0.60, 0.97)	1.26 (0.98, 1.61)
Log transformed (Per unit higher)	0.76 (0.67, 0.87)	0.86 (0.76, 0.98)	0.98 (0.86, 1.12)	1.02 (0.87, 1.20)	1.04 (0.88, 1.23)	0.79 (0.65, 0.95)	0.74 (0.60, 0.92)	0.83 (0.67, 1.02)	1.24 (0.99, 1.56)
Log transformed (Per SD (1.51) higher)	0.66 (0.54, 0.81)	0.80 (0.65, 0.97)	0.97 (0.79, 1.19)	1.04 (0.81, 1.33)	1.06 (0.83, 1.36)	0.70 (0.53, 0.92)	0.64 (0.46, 0.88)	0.75 (0.55, 1.03)	1.39 (0.98, 1.97)

**SHBG nmol/L**									
Q1	1.00 (0.95, 1.05)	1.00 (0.95, 1.05)	1.00 (0.94, 1.06)	1.00 (0.96, 1.04)	1.00 (0.96, 1.04)	1.00 (0.96, 1.05)	1.00 (0.93, 1.07)	1.00 (0.93, 1.07)	1.00 (0.93, 1.08)
Q2	0.80 (0.75, 0.85)	0.78 (0.74, 0.83)	0.97 (0.91, 1.03)	1.03 (0.99, 1.07)	0.88 (0.84, 0.92)	0.98 (0.94, 1.02)	0.77 (0.72, 0.83)	0.89 (0.83, 0.96)	0.99 (0.92, 1.06)
Q3	0.72 (0.68, 0.77)	0.73 (0.69, 0.78)	1.02 (0.96, 1.08)	1.04 (1.00, 1.09)	0.81 (0.78, 0.84)	0.96 (0.92, 1.00)	0.69 (0.64, 0.75)	0.91 (0.84, 0.98)	1.06 (0.99, 1.15)
Q4	0.58 (0.54, 0.62)	0.63 (0.59, 0.68)	0.95 (0.88, 1.02)	1.06 (1.02, 1.11)	0.74 (0.71, 0.77)	0.95 (0.91, 0.99)	0.55 (0.50, 0.59)	0.85 (0.78, 0.92)	1.00 (0.92, 1.09)
Log transformed (Per unit higher)	0.70 (0.66, 0.74)	0.73 (0.69, 0.77)	1.02 (0.95, 1.09)	1.05 (1.00, 1.10)	0.75 (0.72, 0.79)	0.94 (0.89, 0.99)	0.67 (0.62, 0.72)	0.97 (0.89, 1.05)	1.09 (1.00, 1.18)
Log transformed (Per SD (0.51) higher)	0.83 (0.81, 0.86)	0.85 (0.83, 0.88)	1.01 (0.98, 1.05)	1.02 (1.00, 1.05)	0.87 (0.84, 0.89)	0.97 (0.94, 1.00)	0.81 (0.78, 0.85)	0.98 (0.94, 1.02)	1.04 (1.00, 1.09)
**FAI nmol/L**									
Q1	1.00 (0.93 ,1.08)	1.00 (0.93 ,1.08)	1.00 (0.93 ,1.08)	1.00 (0.96 ,1.04)	1.00 (0.96 ,1.04)	1.00 (0.96 ,1.04)	1.00 (0.92 ,1.09)	1.00 (0.92 ,1.09)	1.00 (0.92 ,1.09)
Q2	1.16 (1.09 ,1.24)	1.18 (1.10 ,1.26)	1.05 (0.98 ,1.13)	0.91 (0.87 ,0.94)	1.03 (0.99 ,1.07)	1.03 (0.99 ,1.07)	1.28 (1.19 ,1.39)	1.15 (1.06 ,1.24)	1.02 (0.95 ,1.11)
Q3	1.21 (1.14 ,1.30)	1.25 (1.17 ,1.33)	1.00 (0.94 ,1.07)	0.80 (0.76 ,0.83)	1.05 (1.00 ,1.09)	1.03 (0.99 ,1.07)	1.53 (1.41 ,1.65)	1.19 (1.10 ,1.29)	0.97 (0.90 ,1.05)
Q4	1.29 (1.21 ,1.37)	1.38 (1.29 ,1.47)	0.92 (0.86 ,0.99)	0.72 (0.69 ,0.76)	1.20 (1.15 ,1.26)	1.13 (1.08 ,1.19)	1.78 (1.64 ,1.92)	1.15 (1.06 ,1.24)	0.82 (0.75 ,0.89)
Log transformed (Per unit higher)	1.16 (1.10, 1.22)	1.21 (1.15, 1.27)	0.97 (0.92, 1.02)	0.72 (0.69, 0.76)	1.14 (1.07, 1.21)	1.09 (1.02, 1.15)	1.60 (1.49, 1.72)	1.06 (0.98, 1.15)	0.89 (0.82, 0.97)
Log transformed (Per SD (1.53) higher)	1.26 (1.16, 1.35)	1.34 (1.24, 1.45)	0.95 (0.88, 1.03)	0.61 (0.57, 0.66)	1.22 (1.11, 1.34)	1.14 (1.04, 1.24)	2.06 (1.85, 2.29)	1.10 (0.97, 1.24)	0.84 (0.74, 0.95)

Footnote:

Multiple adjusted models adjusted for age, smoking status, total cholesterol, BMI, Systolic blood pressure, Townsend deprivation score, diabetes, any anti-hypertensive or lipid lowering medication.

O/T = Oestradiol / Testosterone, Free androgen index (FAI), sex hormone–binding globulin (SHBG)

Table S5: Association of combinations of sex hormones with MI by sex

	Women	Women	Women	Men	Men	Men	Women to Men RHR	Women to Men RHR	Women to Men RHR
	**Only sex hormone adjusted**	**Age-adjusted**	**Multiple adjusted**	**Only sex hormone adjusted**	**Age-adjusted**	**Multiple adjusted**	**Only sex hormone adjusted**	**Age-adjusted**	**Multiple adjusted**
Models for Oestradiol									
**Oestradiol + Testosterone**									
Log transformed (Per unit higher)	0.66 (0.57, 0.80)	0.87 (0.74, 1.03)	0.98 (0.83, 1.16)	0.99 (0.73, 1.34)	1.14 (0.84, 1.54)	0.99 (0.71, 1.37)	0.69 (0.48, 0.97)	0.77 (0.54, 1.08)	0.99 (0.69, 1.43)
**Oestradiol + SHBG**									
Log transformed (Per unit higher)	0.72 (0.61, 0.85)	1.02 (0.86, 1.20)	1.05 (0.89, 1.24)	1.10 (0.81, 1.50)	1.28 (0.95, 1.73)	1.11 (0.80, 1.52)	0.65 (0.46, 0.92)	0.79 (0.56, 1.12)	0.95 (0.66, 1.36)
Models for Testosterone									
**Testosterone + Oestradiol**									
Log transformed (Per unit higher)	1.08 (0.88, 1.33)	1.18 (0.97, 1.44)	1.02 (0.83, 1.24)	0.96 (0.79, 1.17)	1.00 (0.82, 1.22)	1.39 (1.12, 1.73)	1.13 (0.84, 1.50)	1.19 (0.90, 1.56)	0.73 (0.54, 0.98)
**Testosterone + SHBG**									
Log transformed (Per unit higher)	0.89 (0.82, 0.95)	1.03 (0.96, 1.11)	0.95 (0.89, 1.02)	0.63 (0.60, 0.67)	0.91 (0.84, 0.97)	1.07 (0.99, 1.15)	1.40 (1.27, 1.54)	1.14 (1.03, 1.26)	0.89 (0.80, 0.99)
Models for SHBG									
**SHBG + oestradiol**									
Log transformed (Per unit higher)	0.89 (0.74, 1.07)	0.72 (0.61, 0.86)	1.00 (0.83, 1.21)	1.09 (0.94, 1.28)	0.79 (0.67, 0.93)	0.96 (0.80, 1.14)	0.81 (0.64, 1.04)	0.92 (0.72, 1.17)	1.05 (0.81, 1.36)
**SHBG + Testosterone**									
Log transformed (Per unit higher)	0.68 (0.64, 0.73)	0.71 (0.66, 0.76)	1.02 (0.94, 1.10)	1.27 (1.20, 1.34)	0.79 (0.74, 0.84)	0.91 (0.86, 0.97)	0.54 (0.49, 0.59)	0.90 (0.82, 0.99)	1.11 (1.01, 1.23)

Footnote:

Multiple adjusted models adjusted for age, smoking status, total cholesterol, BMI, Systolic blood pressure, Townsend deprivation score, diabetes, any anti-hypertensive or lipid lowering medication.

Free androgen index (FAI), sex hormone–binding globulin (SHBG)

Table S6: Association of sex hormones with MI by menopause status (for women)

	Premenopausal			Postmenopausal			Postmenopausal vs Premenopausal RHR		
	Unadjusted	Age-adjusted	Multiple adjusted	Unadjusted	Age-adjusted	Multiple adjusted	Unadjusted	Age-adjusted	Multiple adjusted
**Oestradiol pmol/L**									
Q1	1.00 (0.77, 1.30)	1.00 (0.77, 1.30)	1.00 (0.77, 1.30)	1.00 (0.81, 1.24)	1.00 (0.81, 1.24)	1.00 (0.80, 1.24)	1.00 (0.71, 1.40)	1.00 (0.71, 1.40)	1.00 (0.71, 1.41)
Q2	0.76 (0.58, 0.99)	0.77 (0.59, 1.01)	0.79 (0.60, 1.03)	0.78 (0.56, 1.07)	0.88 (0.64, 1.22)	0.99 (0.72, 1.37)	1.03 (0.67, 1.56)	1.15 (0.76, 1.75)	1.26 (0.83, 1.92)
Q3	0.63 (0.47, 0.83)	0.64 (0.48, 0.85)	0.71 (0.53, 0.94)	0.71 (0.48, 1.06)	0.84 (0.56, 1.25)	0.92 (0.61, 1.39)	1.13 (0.69, 1.84)	1.31 (0.80, 2.14)	1.31 (0.79, 2.15)
Q4	0.80 (0.63, 1.02)	0.79 (0.62, 1.01)	0.91 (0.71, 1.17)	0.84 (0.53, 1.33)	1.14 (0.72, 1.81)	1.28 (0.81, 2.04)	1.04 (0.62, 1.76)	1.44 (0.85, 2.43)	1.41 (0.83, 2.38)
Log transformed (Per unit higher)	0.90 (0.72, 1.12)	0.89 (0.71, 1.11)	0.99 (0.79, 1.24)	0.74 (0.54, 1.03)	0.93 (0.67, 1.28)	0.99 (0.72, 1.38)	0.83 (0.56, 1.23)	1.04 (0.71, 1.55)	0.99 (0.67, 1.47)
Log transformed (Per SD (0.62) higher)	0.93 (0.81, 1.07)	0.93 (0.81, 1.06)	1.00 (0.87, 1.14)	0.83 (0.68, 1.02)	0.95 (0.78, 1.17)	1.00 (0.81, 1.22)	0.89 (0.70, 1.13)	1.03 (0.81, 1.31)	1.01 (0.79, 1.28)
Detectable	1.00 (0.88, 1.14)	1.00 (0.87, 1.15)	1.00 (0.87, 1.15)	1.00 (0.86, 1.17)	1.00 (0.86, 1.17)	1.00 (0.85, 1.17)	1.00 (0.82, 1.22)	1.00 (0.81, 1.23)	1.00 (0.81, 1.24)
Undetectable	1.53 (1.29, 1.81)	1.13 (0.94, 1.35)	0.98 (0.82, 1.18)	1.18 (1.14, 1.22)	0.86 (0.83, 0.89)	0.87 (0.84, 0.90)	0.77 (0.65, 0.92)	0.76 (0.63, 0.92)	0.89 (0.73, 1.07)
Missing	1.06 (0.78, 1.44)	0.96 (0.71, 1.31)	0.97 (0.67, 1.41)	1.11 (1.00, 1.23)	0.82 (0.74, 0.91)	0.80 (0.70, 0.91)	1.05 (0.76, 1.46)	0.86 (0.62, 1.18)	0.82 (0.55, 1.23)
**Testosterone nmol/L**									
Q1	1.00 (0.77, 1.29)	1.00 (0.77, 1.29)	1.00 (0.77, 1.29)	1.00 (0.93, 1.07)	1.00 (0.93, 1.07)	1.00 (0.93, 1.07)	1.00 (0.77, 1.30)	1.00 (0.77, 1.30)	1.00 (0.77, 1.31)
Q2	0.93 (0.73, 1.17)	0.97 (0.76, 1.22)	0.97 (0.76, 1.22)	0.90 (0.83, 0.97)	0.92 (0.85, 0.99)	0.90 (0.83, 0.97)	0.97 (0.76, 1.24)	0.95 (0.74, 1.22)	0.93 (0.73, 1.19)
Q3	1.01 (0.82, 1.24)	1.10 (0.90, 1.36)	1.06 (0.86, 1.30)	0.93 (0.86, 1.01)	0.96 (0.89, 1.04)	0.90 (0.84, 0.98)	0.92 (0.74, 1.14)	0.87 (0.70, 1.08)	0.85 (0.68, 1.06)
Q4	1.10 (0.92, 1.32)	1.24 (1.04, 1.49)	1.08 (0.90, 1.30)	0.96 (0.89, 1.04)	1.00 (0.93, 1.08)	0.90 (0.83, 0.97)	0.87 (0.71, 1.06)	0.11 (0.66, 0.98)	0.83 (0.68, 1.01)
Log transformed (Per unit higher)	1.16 (0.92, 1.48)	1.29 (1.02, 1.64)	1.11 (0.88, 1.41)	0.99 (0.92, 1.08)	1.02 (0.94, 1.11)	0.94 (0.87, 1.02)	0.85 (0.66, 1.10)	0.79 (0.62, 1.02)	0.85 (0.66, 1.09)
Log transformed (Per SD (1.28) higher)	1.22 (0.90, 1.65)	1.39 (1.02, 1.88)	1.14 (0.85, 1.55)	0.99 (0.89, 1.10)	1.03 (0.93, 1.14)	0.93 (0.83, 1.03)	0.81 (0.59, 1.12)	0.74 (0.54, 1.02)	0.81 (0.59, 1.11)
Detectable	1.00 (0.90, 1.11)	1.00 (0.90, 1.11)	1.00 (0.90, 1.11)	1.00 (0.96, 1.04)	1.00 (0.96, 1.04)	1.00 (0.96, 1.04)	1.00 (0.89, 1.12)	1.00 (0.89, 1.12)	1.00 (0.89, 1.12)
Undetectable	1.30 (0.96, 1.78)	1.11 (0.81, 1.52)	1.19 (0.87, 1.63)	1.22 (1.13, 1.31)	1.15 (1.07, 1.24)	1.21 (1.12, 1.30)	0.93 (0.68, 1.28)	1.03 (0.75, 1.42)	1.01 (0.73, 1.40)
Missing	0.77 (0.46, 1.27)	0.76 (0.46, 1.26)	0.55 (0.14, 2.22)	1.04 (0.90, 1.20)	1.03 (0.90, 1.19)	0.85 (0.58, 1.25)	1.37 (0.81, 2.32)	1.36 (0.81, 2.31)	1.53 (0.36, 6.46)
**O/T ratio nmol/L**									
Q1	1.00 (0.77, 1.30)	1.00 (0.77, 1.30)	1.00 (0.77, 1.30)	1.00 (0.76, 1.32)	1.00 (0.75, 1.33)	1.00 (0.73, 1.36)	1.00 (0.68, 1.46)	1.00 (0.68, 1.47)	1.00 (0.67, 1.50)
Q2	0.72 (0.54, 0.96)	0.71 (0.53, 0.94)	0.79 (0.59, 1.06)	1.06 (0.76, 1.48)	1.19 (0.85, 1.67)	1.41 (1.01, 1.97)	1.48 (0.95, 2.30)	1.69 (1.09, 2.63)	1.83 (1.17, 2.85)
Q3	0.73 (0.55, 0.97)	0.71 (0.53, 0.94)	0.85 (0.64, 1.13)	0.84 (0.56, 1.28)	0.99 (0.65, 1.50)	1.16 (0.76, 1.76)	1.16 (0.70, 1.93)	1.40 (0.85, 2.32)	1.38 (0.83, 2.28)
Q4	0.80 (0.62, 1.05)	0.76 (0.58, 1.00)	1.01 (0.77, 1.32)	0.89 (0.56, 1.42)	1.08 (0.68, 1.72)	1.33 (0.84, 2.12)	1.11 (0.65, 1.90)	1.42 (0.83, 2.43)	1.33 (0.78, 2.28)
Log transformed (Per unit higher)	0.87 (0.71, 1.06)	0.85 (0.69, 1.03)	0.98 (0.81, 1.20)	0.90 (0.70, 1.15)	1.01 (0.79, 1.29)	1.12 (0.87, 1.43)	1.03 (0.76, 1.41)	1.20 (0.87, 1.63)	1.13 (0.82, 1.56)
Log transformed (Per SD (1.51) higher)	0.81 (0.60, 1.09)	0.78 (0.58, 1.04)	0.98 (0.72, 1.32)	0.85 (0.59, 1.23)	1.02 (0.70, 1.47)	1.18 (0.81, 1.72)	1.05 (0.65, 1.69)	1.31 (0.82, 2.10)	1.21 (0.74, 1.96)

**SHBG nmol/L**									
Q1	1.00 (0.83, 1.20)	1.00 (0.83, 1.20)	1.00 (0.81, 1.24)	1.00 (0.94, 1.07)	1.00 (0.94, 1.07)	1.00 (0.93, 1.07)	1.00 (0.82, 1.22)	1.00 (0.82, 1.22)	1.00 (0.80, 1.25)
Q2	0.63 (0.51, 0.79)	0.65 (0.52, 0.81)	0.91 (0.74, 1.12)	0.85 (0.79, 0.91)	0.82 (0.76, 0.88)	1.00 (0.94, 1.07)	1.34 (1.07, 1.68)	1.26 (1.00, 1.58)	1.10 (0.88, 1.37)
Q3	0.50 (0.40, 0.63)	0.53 (0.42, 0.66)	0.88 (0.70, 1.10)	0.81 (0.76, 0.87)	0.79 (0.73, 0.85)	1.07 (1.00, 1.16)	1.61 (1.27, 2.04)	1.50 (1.18, 1.89)	1.22 (0.97, 1.55)
Q4	0.35 (0.28, 0.45)	0.37 (0.29, 0.47)	0.69 (0.53, 0.89)	0.69 (0.63, 0.75)	0.67 (0.62, 0.73)	0.99 (0.91, 1.08)	1.94 (1.51, 2.51)	1.82 (1.41, 2.35)	1.44 (1.10, 1.89)
Log transformed (Per unit higher)	0.48 (0.39, 0.58)	0.49 (0.41, 0.60)	0.82 (0.65, 1.02)	0.78 (0.73, 0.84)	0.76 (0.70, 0.81)	1.05 (0.97, 1.14)	1.64 (1.33, 2.02)	1.53 (1.24, 1.89)	1.29 (1.01, 1.63)
Log transformed (Per SD (0.51) higher)	0.69 (0.62, 0.76)	0.70 (0.63, 0.77)	0.90 (0.80, 1.01)	0.88 (0.85, 0.92)	0.87 (0.84, 0.90)	1.03 (0.98, 1.07)	1.28 (1.15, 1.43)	1.24 (1.12, 1.38)	1.14 (1.01, 1.28)
**FAI nmol/L**									
Q1	1.00 (0.75, 1.34)	1.00 (0.75, 1.34)	1.00 (0.74, 1.35)	1.00 (0.92, 1.09)	1.00 (0.92, 1.09)	1.00 (0.91, 1.09)	1.00 (0.74, 1.35)	1.00 (0.74, 1.35)	1.00 (0.73, 1.36)
Q2	1.28 (1.00, 1.65)	1.31 (1.02, 1.69)	1.18 (0.92, 1.52)	1.17 (1.08, 1.27)	1.18 (1.09, 1.27)	1.06 (0.98, 1.14)	0.92 (0.70, 1.19)	0.90 (0.69, 1.16)	0.90 (0.69, 1.17)
Q3	1.83 (1.49, 2.26)	1.91 (1.55, 2.35)	1.45 (1.18, 1.78)	1.21 (1.12, 1.31)	1.23 (1.14, 1.33)	1.00 (0.92, 1.08)	0.66 (0.53, 0.83)	0.64 (0.51, 0.80)	0.69 (0.55, 0.85)
Q4	2.32 (1.92, 2.79)	2.46 (2.04, 2.96)	1.37 (1.11, 1.68)	1.22 (1.13, 1.32)	1.28 (1.19, 1.38)	0.88 (0.81, 0.96)	0.53 (0.43, 0.64)	0.52 (0.43, 0.64)	0.64 (0.52, 0.80)
Log transformed (Per unit higher)	1.61 (1.37, 1.89)	1.66 (1.42, 1.95)	1.17 (0.98, 1.39)	1.12 (1.06, 1.19)	1.16 (1.09, 1.23)	0.94 (0.88, 1.00)	0.70 (0.59, 0.83)	0.70 (0.59, 0.83)	0.80 (0.67, 0.96)
Log transformed (Per SD (0.51) higher)	2.07 (1.62, 2.65)	2.17 (1.70, 2.78)	1.27 (0.97, 1.65)	1.19 (1.09, 1.30)	1.25 (1.14, 1.37)	0.90 (0.82, 1.00)	0.58 (0.44, 0.75)	0.57 (0.44, 0.75)	0.71 (0.54, 0.94)

Footnote:

Multiple adjusted models adjusted for age, smoking status, total cholesterol, BMI, Systolic blood pressure, Townsend deprivation score, diabetes, any anti-hypertensive or lipid lowering medication.

O/T = Oestradiol / Testosterone, Free androgen index (FAI), sex hormone–binding globulin (SHBG)

Table S7: Association of sex hormones with MI by menopause status and HRT use (for women)

	Pre-menopausal		Post-menopausal	
	**HRT use - no**	**HRT use - yes**	**HRT use - no**	**HRT use - yes**
N	*59814*	*2279*	*84613*	*74751*
N (MI events)	*352*	*37*	*1606*	*1936*
	**HR (95%CI)**	**HR (95%CI)**	**HR (95%CI)**	**HR (95%CI)**
**Oestradiol pmol/L - Log transformed (Per unit higher)**				
Crude	0.90 (0.72, 1.13)	0.89 (0.31, 2.53)	0.47 (0.22, 0.98)	0.84 (0.57, 1.22)
Age-adjusted	0.88 (0.70, 1.11)	0.93 (0.32, 2.69)	0.48 (0.22, 1.04)	0.99 (0.68, 1.45)
Multiple adjusted	0.99 (0.79, 1.24)	1.22 (0.41, 3.61)	0.57 (0.26, 1.26)	1.00 (0.69, 1.45)
**Testosterone nmol/L - Log transformed (Per unit higher)**				
Crude	1.13 (0.88, 1.46)	2.14 (0.97, 4.70)	1.01 (0.90, 1.14)	1.01 (0.91, 1.13)
Age-adjusted	1.22 (0.95, 1.57)	2.70 (1.19, 6.11)	1.03 (0.91, 1.16)	1.04 (0.93, 1.16)
Multiple adjusted	1.04 (0.81, 1.33)	2.35 (1.02, 5.42)	0.93 (0.83, 1.05)	0.96 (0.86, 1.07)
**O/T ratio nmol/L - Log transformed (Per unit higher)**				
Crude	0.89 (0.72, 1.08)	0.48 (0.18, 1.30)	1.03 (0.65, 1.62)	0.78 (0.58, 1.06)
Age-adjusted	0.86 (0.70, 1.05)	0.47 (0.17, 1.29)	1.12 (0.70, 1.79)	0.86 (0.65, 1.16)
Multiple adjusted	1.01 (0.82, 1.23)	0.60 (0.23, 1.57)	1.36 (0.81, 2.28)	0.92 (0.68, 1.23)
**SHBG nmol/L - Log transformed (Per unit higher)**				
Crude	0.45 (0.37, 0.55)	0.81 (0.43, 1.56)	0.69 (0.62, 0.77)	0.85 (0.78, 0.94)
Age-adjusted	0.47 (0.38, 0.57)	0.86 (0.45, 1.66)	0.67 (0.60, 0.74)	0.84 (0.76, 0.93)
Multiple adjusted	0.77 (0.61, 0.98)	1.45 (0.71, 2.96)	0.96 (0.85, 1.09)	1.11 (0.99, 1.24)
**FAI nmol/L - Log transformed (Per unit higher)**				
Crude	1.62 (1.37, 1.92)	1.75 (1.03, 2.95)	1.20 (1.11, 1.31)	1.08 (1.00, 1.17)
Age-adjusted	1.66 (1.40, 1.96)	1.83 (1.07, 3.16)	1.24 (1.14, 1.35)	1.10 (1.01, 1.19)
Multiple adjusted	1.15 (0.96, 1.39)	1.27 (0.70, 2.29)	0.97 (0.88, 1.06)	0.92 (0.84, 1.00)

Footnotes:

Multiple adjusted models adjusted for age, smoking status, total cholesterol, BMI, Systolic blood pressure, Townsend deprivation score, diabetes, any anti-hypertensive or lipid lowering medication.

O/T = Oestradiol / Testosterone, Free androgen index (FAI), sex hormone–binding globulin (SHBG), Hormone Replacement Therapy (HRT)

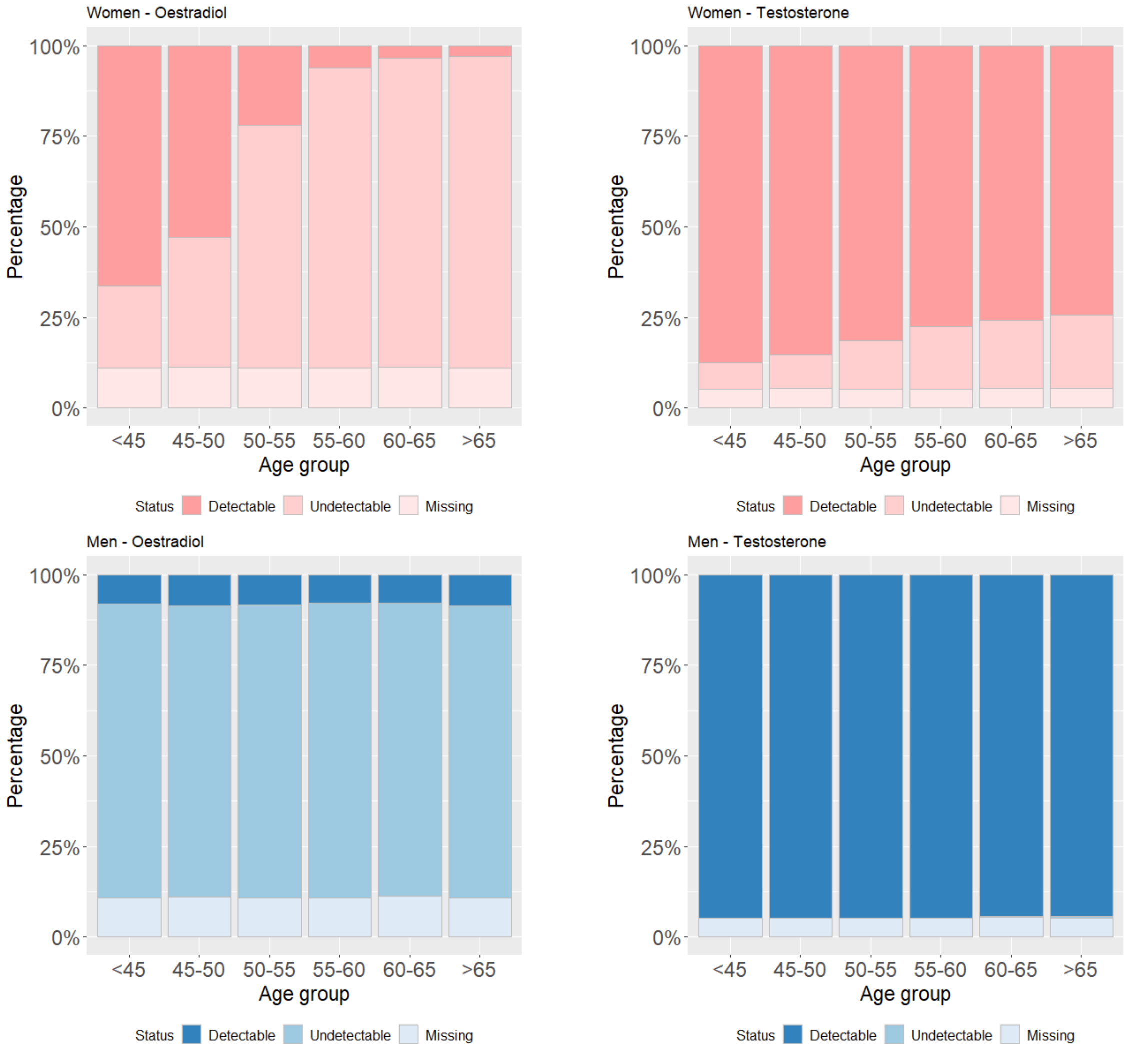

Figure S1: Percentage of Oestrogen and Testosterone detectable status by age group and sex

Pink bars represent women and blue represent men.

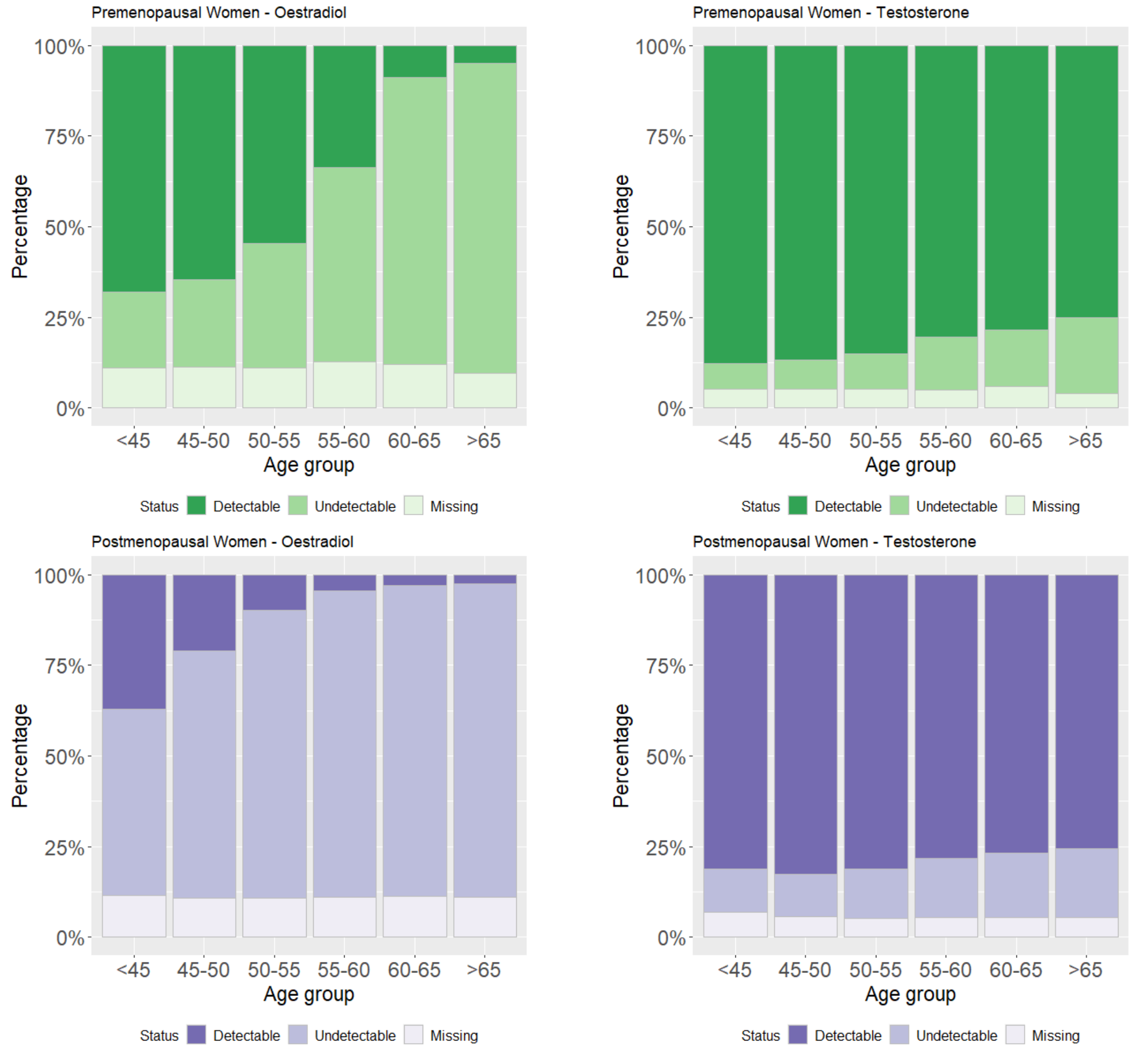

Figure S2: Percentage of Oestrogen and Testosterone detectable status for women by age group and menopause status

Green bars represent premenopausal women and purple for post-menopausal women.

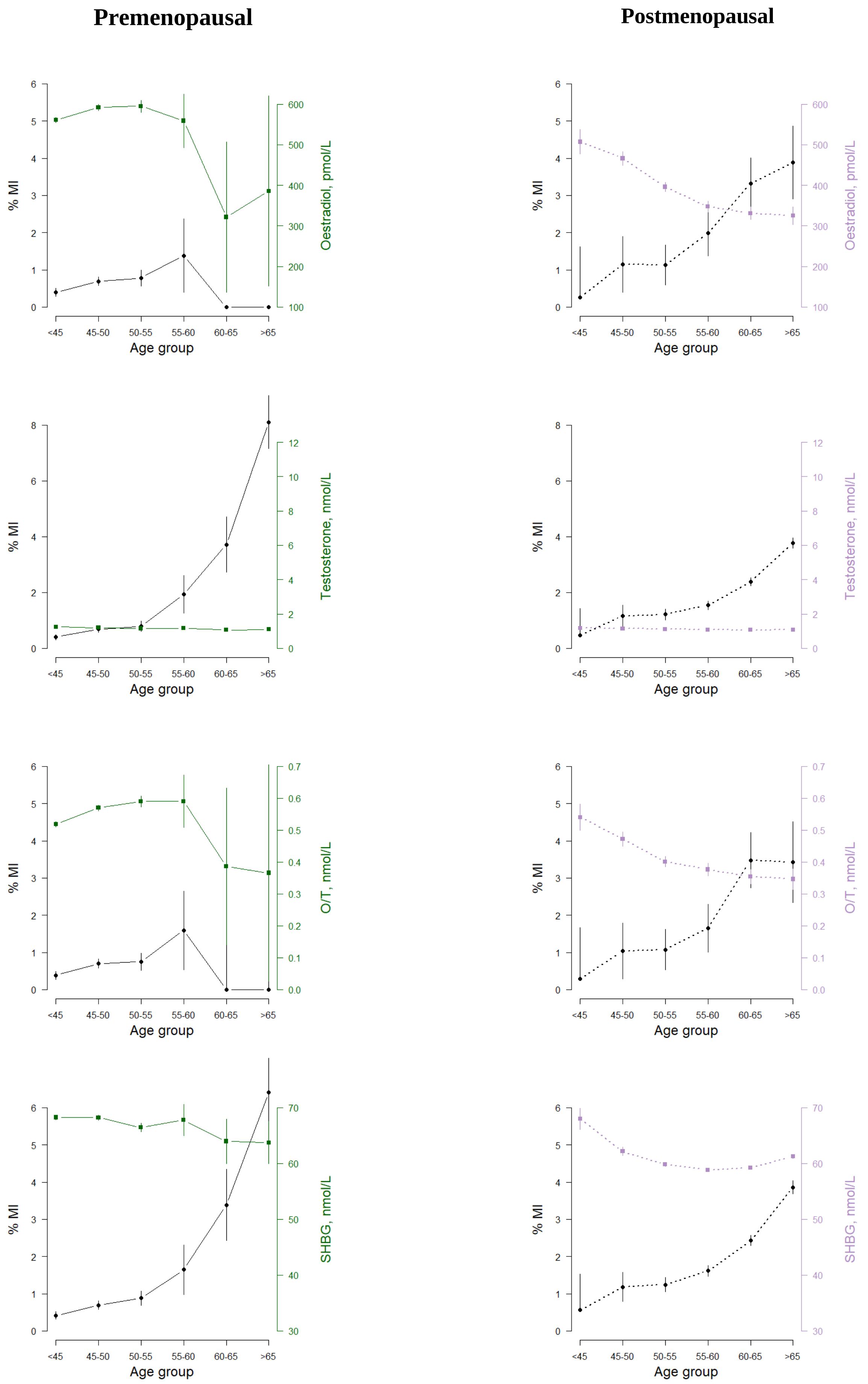
Premenopausal
Postmenopausal
% MI
Oestradiol, pmol/L
Testosterone, nmol/L
O/T, nmol/L
SHBG, nmol/L
Age group
<45
45-50
50-55
55-60
60-65
>65

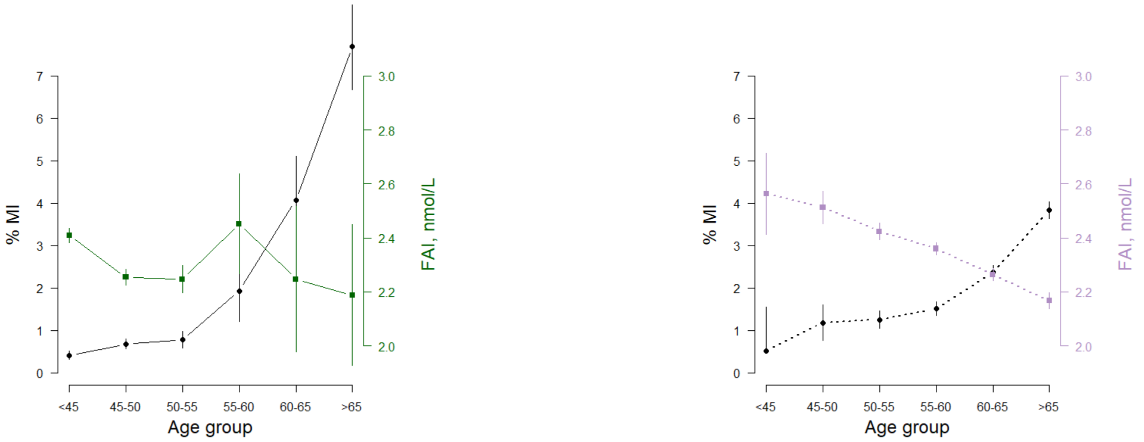

Figure S3: Rates of myocardial infarction and levels of sex hormones, with 95% confidence intervals, by menopause status (for women) and age group.

Footnote: Dark green lines represent sex hormone levels for pre-menopausal women and purple (dotted) for post-menopausal women. Black solid lines represent % MI by age group for pre-menopausal women and black dotted lines for post-menopausal women. Where lower confidence intervals were negative for the % MI these have not been plotted.

O/T = Oestradiol / Testosterone, Free androgen index (FAI), sex hormone–binding globulin (SHBG)

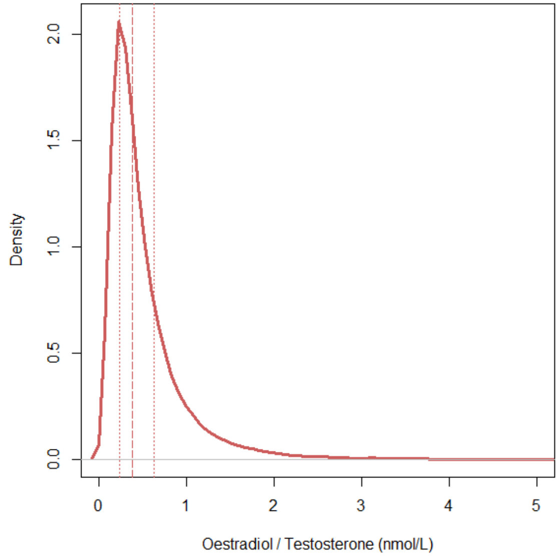

Density plot of Oestradiol / Testosterone (nmol/L) for women. Plotting range of Oestradiol / Testosterone (nmol/L) has been truncated at 5 for plotting purposes, noting that 42 women have Oestradiol / Testosterone (nmol/L) greater than 5.

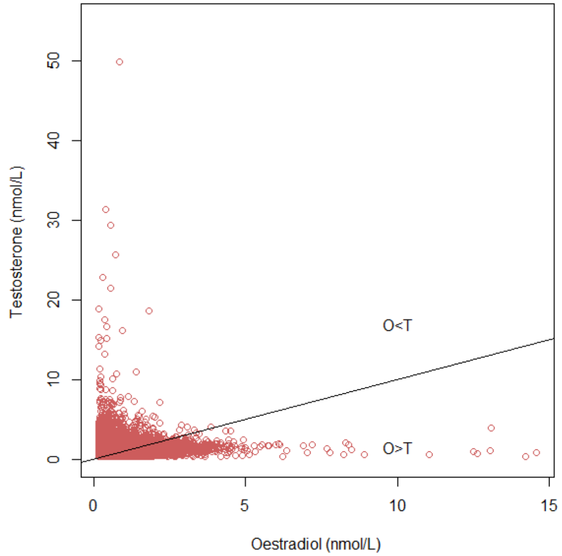

Scatter plot of Oestrogen vs Testosterone in women

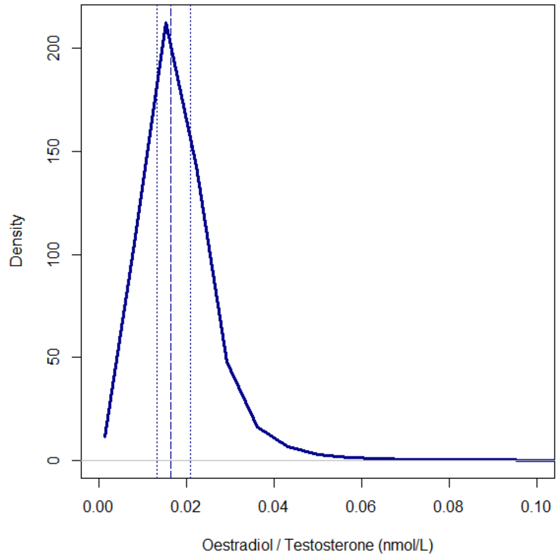

Density plot of Oestradiol / Testosterone (nmol/L) for men Plotting range of Oestradiol / Testosterone (nmol/L) has been truncated at 0.10 for plotting purposes, noting that 44 men have Oestradiol / Testosterone (nmol/L) greater than 0.10.

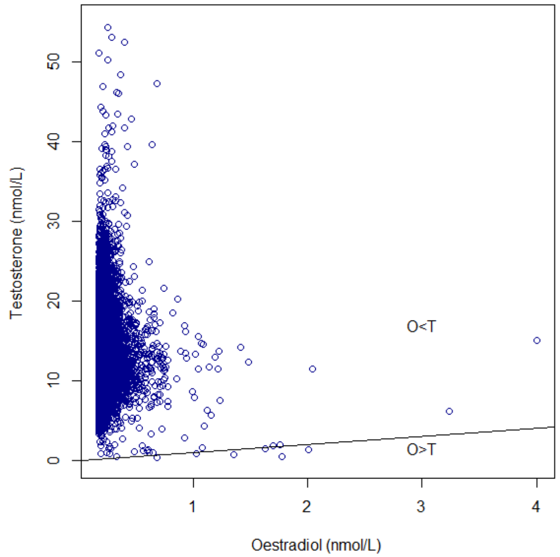

Scatter plot of Oestrogen vs Testosterone in men

Figure S4: Plots of Oestradiol / Testosterone ratio (nmol/L) in women and men

Footnote (Figure S4):

Lower values of the O/T ratio were observed if oestrogen values were lower than testosterone values, and higher values if oestrogen was higher than testosterone. Of the 52,529 women with detectable levels of the O/T ratio, there were 5,522 that had oestrogen concentration greater than testosterone concentration. In men the O/T ratio was generally low, since men tend to have a much higher testosterone level than oestrogen level. Of the 17,580 men with detectable levels of the O/T ratio, six had greater oestrogen concentration than their testosterone concentration.

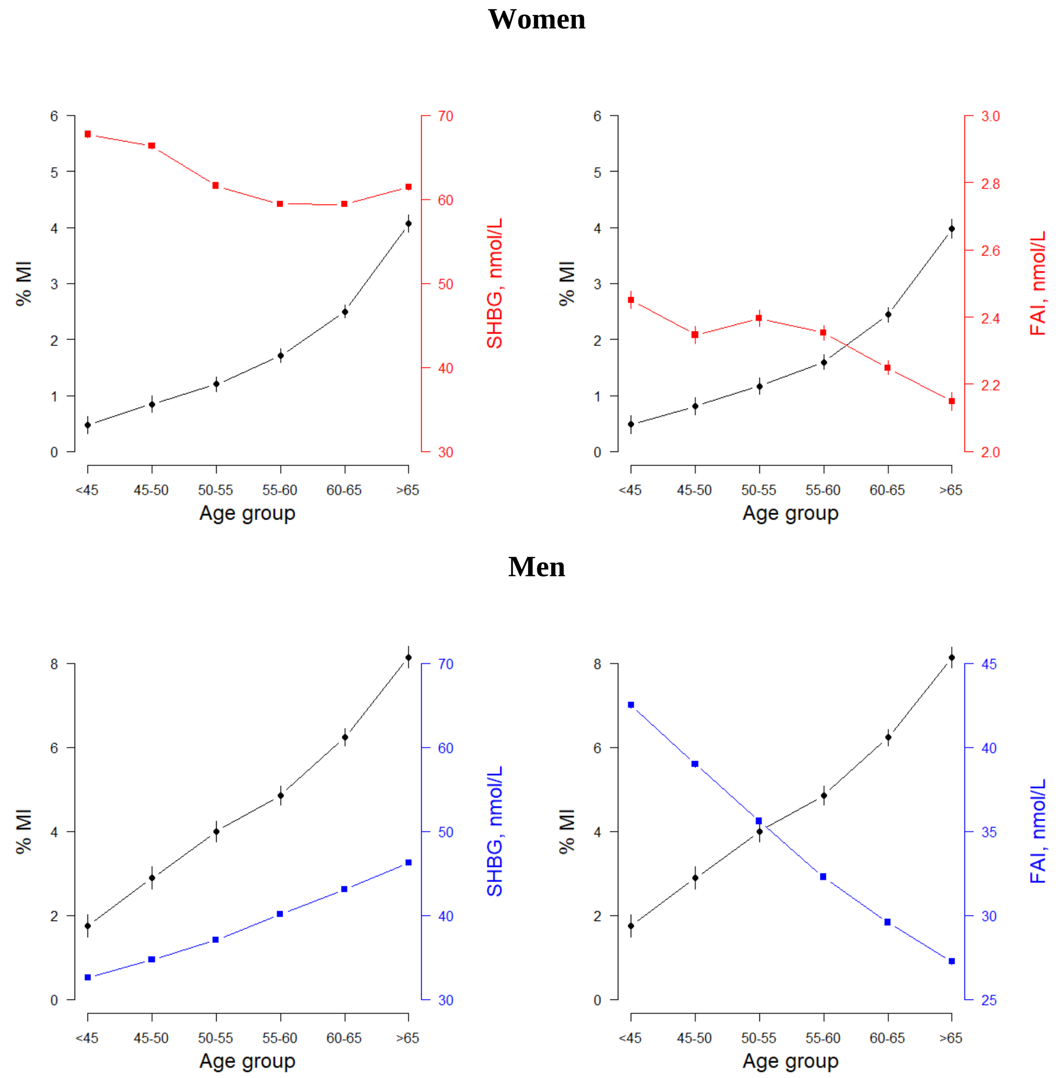

Figure S5: Rates of myocardial infarction and levels of SHBG, and free androgen index (FAI), with 95% confidence intervals, by sex and age group.

Sex hormone–binding globulin (SHBG), Free androgen index (FAI) = Total Testosterone/SHBG x 100

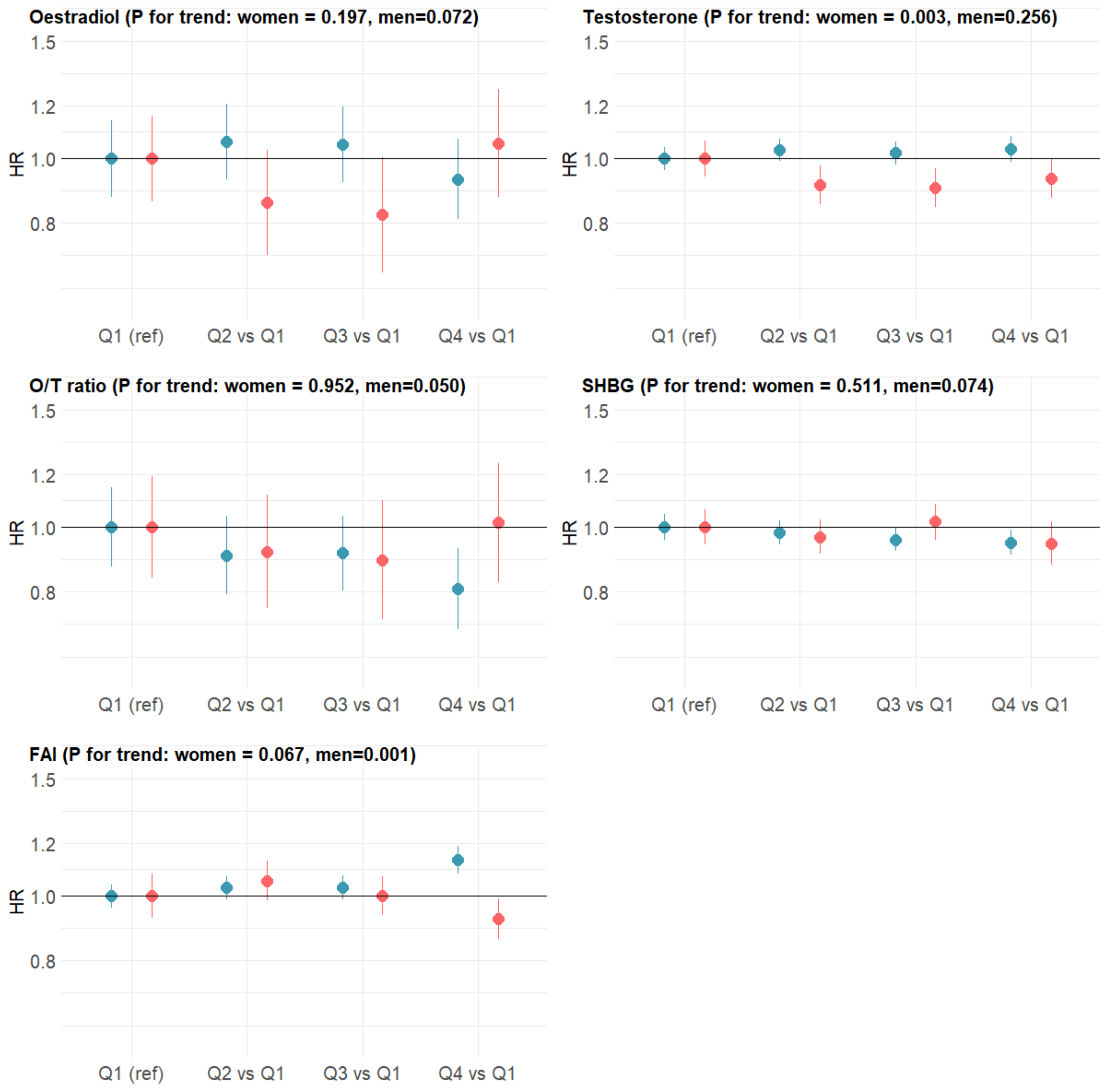

Figure S6: Association of quarters of sex hormones with MI by sex

Multiple adjusted hazard ratios (HR) with 95% confidence intervals: Blue = Men, Pink/red = Women. Multiple adjusted models adjusted for age, smoking status, total cholesterol, BMI, Systolic blood pressure, Townsend deprivation score, diabetes, any anti-hypertensive or lipid lowering medication. O/T = Oestradiol / Testosterone, Free androgen index (FAI), sex hormone–binding globulin (SHBG).

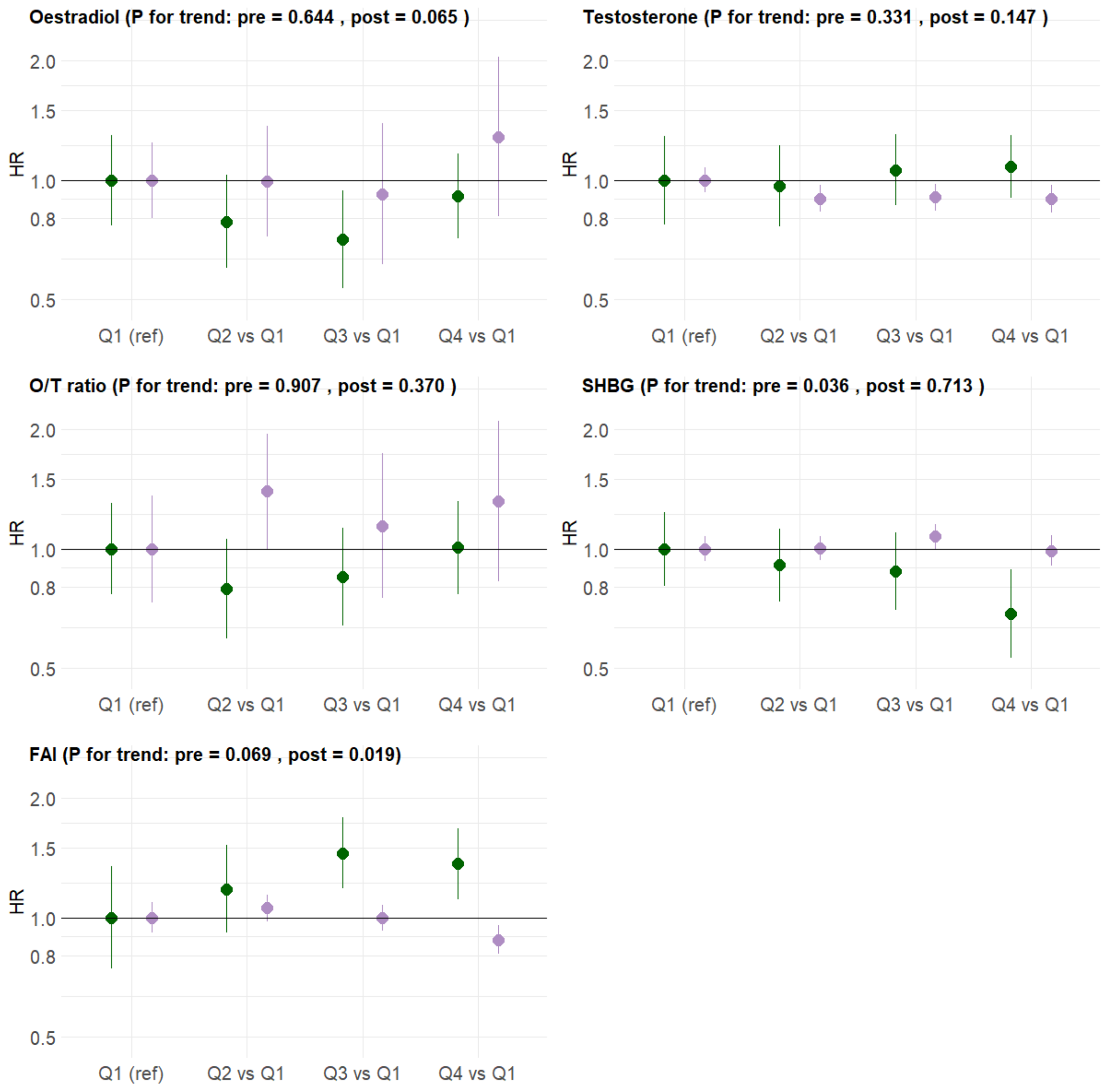

Figure S7: Association of quarters of sex hormones with MI by menopause status (for women)

Multiple adjusted hazard ratios (HR) with 95% confidence intervals: Dark green = pre menopause, purple = post-menopause. Multiple adjusted models adjusted for age, smoking status, total cholesterol, BMI, Systolic blood pressure, Townsend deprivation score, diabetes, any anti-hypertensive or lipid lowering medication. O/T = Oestradiol / Testosterone, Free androgen index (FAI), sex hormone–binding globulin (SHBG)
